# Zn(TFSI)_2_-Mediated Ring-Opening Polymerization for Electrolyte Engineering Toward Stable Aqueous Zinc Metal Batteries

**DOI:** 10.1007/s40820-025-01649-9

**Published:** 2025-01-28

**Authors:** Zhenjie Liu, Murong Xi, Rui Sheng, Yudai Huang, Juan Ding, Zhouliang Tan, Jiapei Li, Wenjun Zhang, Yonggang Wang

**Affiliations:** 1 State Key Laboratory of Chemistry and Utilization of Carbon Based Energy Resources, College of Chemistry, Xinjiang University, Urumqi, 830017 People’s Republic of China; 2 Department of Materials Science and Engineering & Center of Super-Diamond and Advanced Films, City University of Hong Kong, 83 Tat Chee Avenue, Kowloon, Hong Kong SAR People’s Republic of China; 3 Department of Chemistry and Shanghai Key Laboratory of Molecular Catalysis and Innovative Materials, Institute of New Energy, iChEM (Collaborative Innovation Center of Chemistry for Energy Materials), Fudan University, Shanghai, 200433 People’s Republic of China

**Keywords:** Electrolyte engineering, Ring-opening polymerization, Lewis acid catalyst, Zn metal battery

## Abstract

Practical Zn metal batteries have been hindered by several challenges, including Zn dendrite growth, undesirable side reactions, and unstable electrode/electrolyte interface. These issues are particularly more serious in low-concentration electrolytes. Herein, we design a Zn salt-mediated electrolyte with in situ ring-opening polymerization of the small molecule organic solvent. The Zn(TFSI)_2_ salt catalyzes the ring-opening polymerization of (1,3-dioxolane (DOL)), generating oxidation-resistant and non-combustible long-chain polymer (poly(1,3-dioxolane) (pDOL)). The pDOL reduces the active H_2_O molecules in electrolyte and assists in forming stable organic–inorganic gradient solid electrolyte interphase with rich organic constituents, ZnO and ZnF_2_. The introduction of pDOL endows the electrolyte with several advantages: excellent Zn dendrite inhibition, improved corrosion resistance, widened electrochemical window (2.6 V), and enhanced low-temperature performance (freezing point =  − 34.9 °C). Zn plating/stripping in pDOL-enhanced electrolyte lasts for 4200 cycles at 99.02% Coulomb efficiency and maintains a lifetime of 8200 h. Moreover, Zn metal anodes deliver stable cycling for 2500 h with a high Zn utilization of 60%. A Zn//VO_2_ pouch cell assembled with lean electrolyte (electrolyte/capacity (E/C = 41 mL (Ah)^−1^) also demonstrates a capacity retention ratio of 92% after 600 cycles. These results highlight the promising application prospects of practical Zn metal batteries enabled by the Zn(TFSI)_2_-mediated electrolyte engineering.

**Supplementary Information:**

The online version contains supplementary material available at 10.1007/s40820-025-01649-9.

## Highlights

- A novel electrolyte enabled by Zn(TFSI)_2_-mediated ring-opening polymerization strategy for highly reversible aqueous zinc metal batteries was proposed.
- The novel electrolyte has good antioxidant stability and non-inflammability.
- The novel electrolyte widens the electrochemical window, improves the low-temperature performance, and inhibits Zn dendrite. The Zn metal anode shows an 8200 h lifespan at 1 mAh cm^−2^ and a 2500 h lifespan at 60% depth of discharge.

**Supplementary Information:**

The online version contains supplementary material available at 10.1007/s40820-025-01649-9.

## Graphical abstract

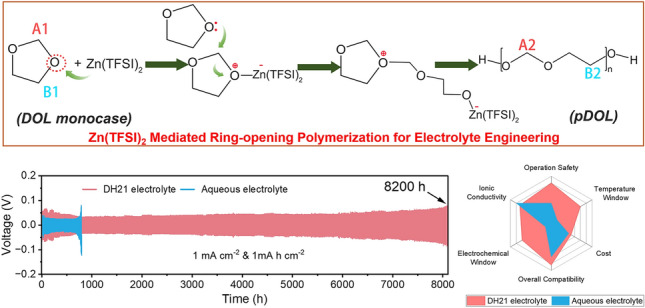

## Introduction

Zn metal batteries have the advantages of high theoretical specific capacity, low cost, and high safety, making them a promising alternative to lithium-ion batteries for electrochemical energy storage applications [1]. Though the studies of Zn metal batteries in the laboratory have demonstrated enhanced cycle life, energy density, and power density, the performance of Zn metal batteries in practical settings has not met expectations [2, 3]. The main bottlenecks constraining the use of Zn metal batteries in practical applications include (i) Zn dendrite growth which leads to internal short circuits and safety issues [3]; (ii) low utilization of Zn metal anode, giving rise to reduced capacity and energy density [4, 5]; (iii) poor electrolyte stability that results in low Coulomb efficiency (CE), cell bulking, and the formation of passivation products on the Zn surface. These side effects are more severe under practical conditions, such as high depth of discharge (DOD), low negative/positive (N/P) ratios, and intermittent conditions [4, 6]; and (iv) high concentration and high dosage of electrolyte that significantly increases the battery cost and reduces the overall energy density [7].

The rational design of the electrolyte solvation structure is one of the key means to simultaneously address these challenges [8–11]. H_2_O in the electrolyte is a double-edged sword: It ensures the safety and excellent ion transport kinetics, but its low thermodynamic stability and highly dynamic and disordered solvation structure around Zn^2+^ can lead to electrolyte decomposition and formation of by-products [12]. These by-products are often loose and difficult to transport ions, ultimately deteriorating battery performance. Therefore, to achieve high capacity and long life of Zn metal batteries, it is necessary to precisely control the amount of free H_2_O in the electrolyte and regulate the solvation structure of Zn^2+^. At the same time, it is also essential to accurately regulate the structure and composition of solid electrolyte interphase (SEI) to meet the needs of practical Zn metal batteries for long life and high-energy density [13, 14].

The use of lightweight, non-flammable, and low-cost H_2_O-organic co-solvent low-concentration electrolytes with improved solvation structure and reduced H_2_O activity can enhance the stability of the Zn anode/electrolyte interface, promote uniform dendrite-free Zn deposition, and build an efficient SEI [15]. Importantly, this approach has the additional properties of reducing electrolyte concentration, weight, and cost, and widening the operating temperature of the electrolyte, which is expected to be favorable for developing practical Zn metal batteries [16–19]. Cui et al. [17] reported a hybrid electrolyte of propylene carbonate (PC) and H_2_O. The modified Zn^2+^ solvation structure promotes the reduction of anions and forms a hydrophobic SEI, which improves the reversibility of the Zn anode. Lu et al. [18] reported a strategy that sacrifices solvated shells to repel active H_2_O molecules. This strategy facilitates the formation of a stable fluorine-rich organic–inorganic gradient SEI at the electrode/electrolyte interface, improving H_2_O corrosion and inhibiting hydrogen evolution rates of the Zn anode. In principle, the ideal H_2_O-organic co-solvent electrolyte should have a wide electrochemical window, high ionic conductivity, and non-flammability. However, this task remains a vast challenge, as the major limitations are mainly rooted in the electrochemical stability and flammability of small molecule organic solvents.

In this work, we design a Zn(TFSI)_2_-mediated electrolyte with in situ ring-opening polymerization of a small molecule organic solvent (1,3-dioxolane (DOL)). The Lewis acidic Zn(TFSI)_2_ plays a dual role; providing the necessary Zn^2+^ and catalyzing the ring-opening polymerization of the cyclic DOL. A co-solvent electrolyte composed of liquid long-chain polymer molecules and H_2_O is successfully prepared without the introduction of other external catalysts. The ring-opening polymerization and reconstruction of DOL monocase increase the intrinsic stability of the electrolyte at high voltage and make it non-inflammability. The electrochemical stability window is widened to 2.6 V with a low salt concentration (0.5 M). The electrolyte also has a low freezing point of − 34.9 °C. Moreover, this electrolyte system can effectively passivate the Zn metal anode and make the SEI rich in inorganic fluoride and polyether derivative fragments, enabling excellent Zn anode/electrolyte interface stability and Zn dendrite inhibition (ACE = 99.02% for 4200 cycles at 1 mA cm^−2^, with a cyclic life more than 8200 h for Zn//Zn symmetric battery). It is worth noting that the SEI film can reduce the interfacial turbulence to stabilize the Zn^2+^ flux of the deep circulating Zn anode and improve the performance of the full battery. A Zn//VO_2_ pouch cell assembled with lean electrolyte (electrolyte/capacity (E/C = 41 mL (Ah)^−1^)) demonstrates a capacity retention ratio of 92% after 600 cycles. This work demonstrates a new strategy to design multifunctional electrolytes for practical Zn metal batteries.

## Results and Discussion

### Zn(TFSI)_2_-Mediated Ring-Opening Polymerization of DOL

**Fig. 1:**
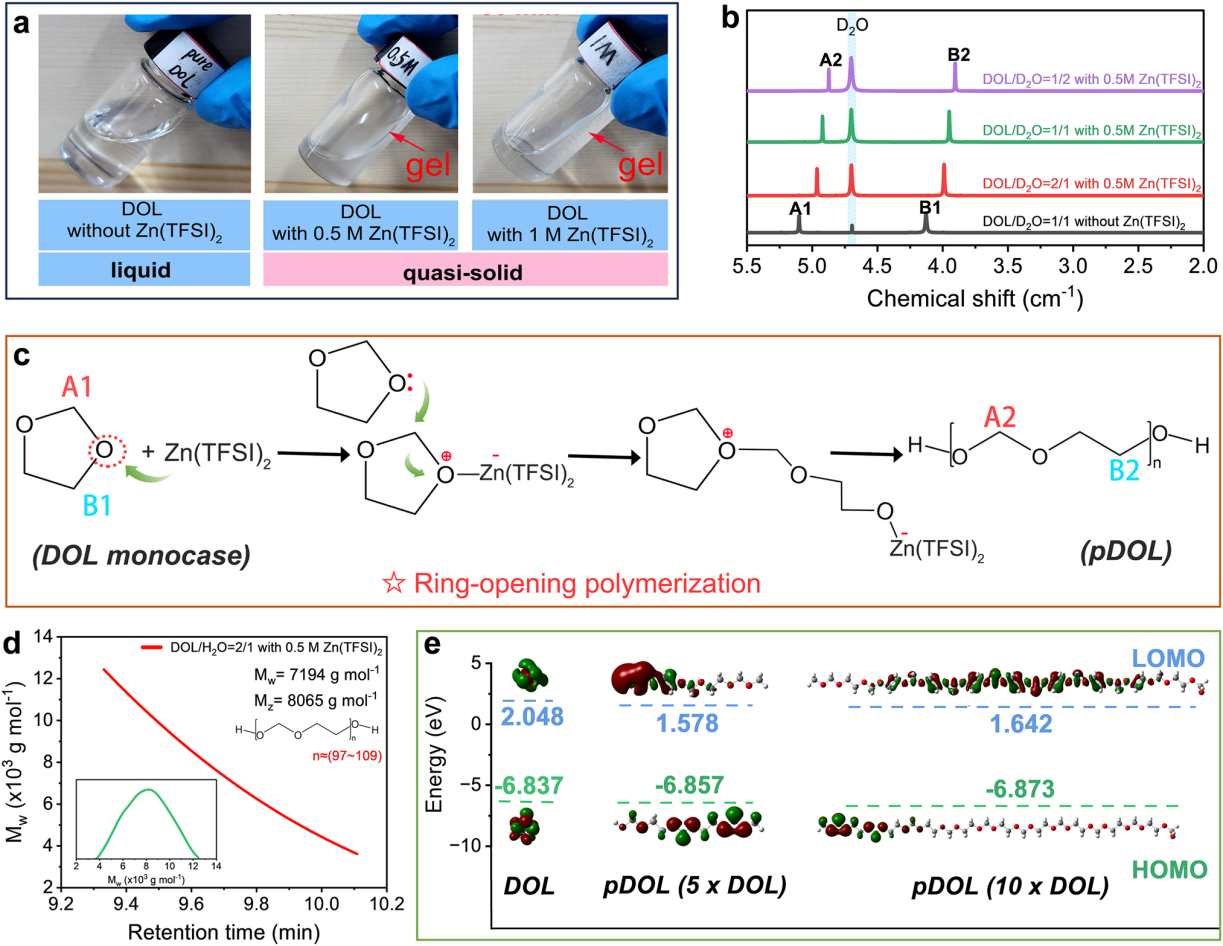
**a** Optical photographs of ring-opening polymerization of DOL induced by Zn(TFSI)_2_. **b** ^1^H NMR spectra of different proportions DOL/D_2_O solutions with/without Zn(TFSI)_2_. **c** Mechanism of ring-opening polymerization of DOL induced by Zn(TFSI)_2_. **d** GPC results after DOL polymerization at DOL/H_2_O = 2/1. **e** Calculated HOMO and LUMO of DOL and pDOL

Due to the unsaturated chemical structure of DOL solvent, it can undergo ring-opening polymerization under the induction of Lewis acid. In this work, we introduce Zn(TFSI)_2_ to catalyze the ring-opening polymerization of DOL monocase. A series of solutions consisting of different concentrations of only Zn(TFSI)_2_ and DOL (i.e., 0.5 and 1.0 M Zn(TFSI)_2_ dissolved in DOL) for gelation experiments. It was observed that the solution gelated after adding Zn(TFSI)_2_ to the DOL solution for 30 min (Fig. 1a).

To further prove that the obtained gel is the polymerized product of DOL (pDOL), ^1^H nuclear magnetic resonance (^1^H NMR) tests were performed (Fig. 1b). In ^1^H NMR spectra, the peaks at chemical shifts of 4.12 (B1) and 5.10 (A1) ppm are the hydrogen of the corresponding structure in DOL. The chemical shift decreases after DOL polymerization, corresponding to the hydrogen in the –O–CH_2_–CH_2_–O–group (B2) and –O–CH_2_–O–group (A2) in pDOL [20]. Figure 1c shows the basic chemical process of Zn(TFSI)_2_-induced DOL polymerization. The positively charged Lewis acid site of Zn(TFSI)_2_ interacts strongly with the oxygen atoms in the DOL, which causes nearby carbon atoms in the DOL to be more positively charged due to the electron-withdrawing effect. Subsequently, the electronegative oxygen atom in another DOL molecule attacks the electropositive carbon atom in the adsorbed DOL, breaking the C-O bond of the DOL and completing the ring-opening process. The above nucleophilic addition reaction is then repeated several times to complete the polymerization and produce the growth molecular chain of pDOL. The electropositive Lewis acid site is the first to trigger the gel reaction, so the spontaneous gel reaction is a typical cationic polymerization [21].

Figure 1d shows the results of gel permeation chromatography (GPC) experiments, which further clarify the polymer properties of pDOL gel. According to the GPC results, the M_w_ of pDOL in DH21 electrolytes is 7194 g mol^−1^ (degree of polymerization is 97 ~ 107), which further clarifies the polymer properties of pDOL gel. In summary, pDOL is formed by ring-opening polymerization of DOL monocase under the catalysis of Zn(TFSI)_2_. In general, the ideal electrolyte should have the low highest occupied molecular orbital (HOMO) to obtain a wide voltage stability window. Figure 1e shows that compared with DOL, the HOMO value of pDOL is lower, and the HOMO value gradually decreases with the increase in polymerization degree, indicating that the increase in ring-opening polymerization degree of DOL will improve the antioxidant stability of the electrolyte. As can be seen from the molecular surface electrostatic potential (ESP) diagram of DOL and pDOL (Fig. S1), the more neatly arranged polymer chains can provide a denser negative potential distribution, which can increase the Zn^2+^ binding sites. By regulating the polymerization of DOL, the problem of poor electrochemical oxidation resistance of organic small molecule solvent is settled, and the path of continuous migration of Zn^2+^ is shorter, which is expected to improve the working voltage and rate performance of the electrolyte [20].

### Electrolyte Structure and Performance Evolution

**Fig. 2:**
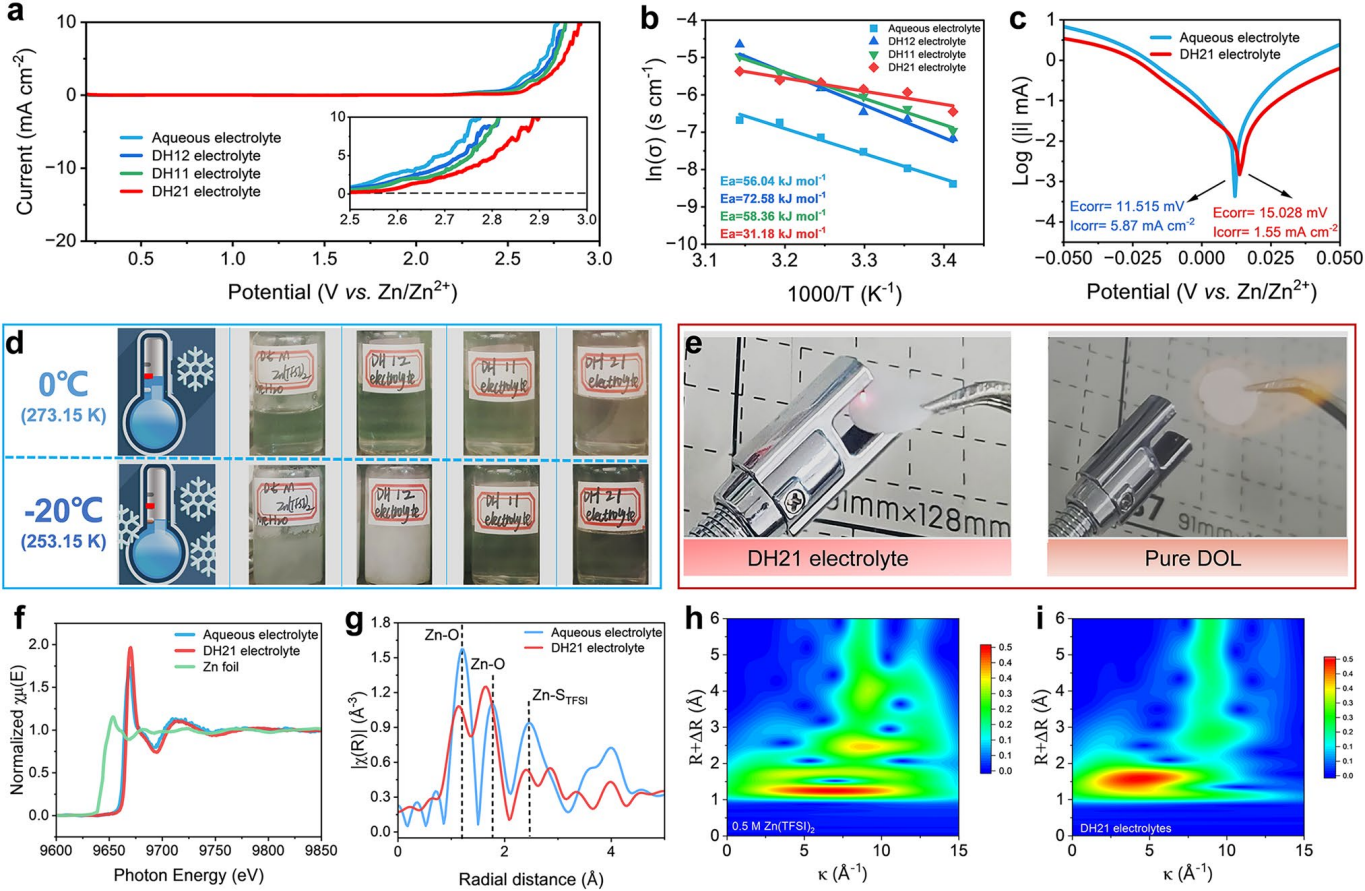
**a** LSV curves of Zn//Cu asymmetric cells and **b** plots of ln(δ) against 1000/T for various electrolytes. **c** Tafel plots of Zn in aqueous electrolyte and DH21 electrolyte (working electrode: Zn foil, counter electrode: Ti foil, and reference electrode: Zn foil). **d** Snapshots of various electrolytes at 0 and − 20 °C. **e** Flammability test of separator soaked with DH21 electrolyte and pure DOL solvent. **f**, **g** Normalized X-ray absorption near-edge structure (XANES) spectra and the corresponding extended X-ray absorption fine structure (EXAFS) spectra of both electrolytes, respectively. Wavelet transform (WT) images for the EXAFS signals of **h** aqueous electrolyte and **i** DH21 electrolyte

As shown in Fig. 2a, the stability of the electrolyte composed of pDOL after solvent molecular reconstruction is significantly improved (the initial voltage of oxidation decomposition of DH21 electrolyte is 2.6 V), but the ionic conductivity decreases slightly with increased pDOL content (Fig. S2). However, it is worth noting that the resistance of aqueous electrolytes with low concentration is significantly affected by temperature. In contrast, the temperature sensitivity is effectively improved with the introduction of pDOL, which is expected to broaden the broad temperature applicability of this electrolyte (Fig. S3, the fitting results are shown in Table S1). Based on the Arrhenius diagram shown in Fig. 2b, the temperature-dependent charge transfer resistance shows that DH21 electrolyte has a low-energy barrier of 31.18 kJ mol^−1^, indicating its rapid desolvation ability. The Zn^2+^ migration number in aqueous electrolyte containing 0.5 M Zn(TFSI)_2_ calculated in Fig. S4 is 0.24, while that in DH21 electrolyte is 0.40, which indicates that pDOL is favorable to the migration of Zn^2+^. The increased migration number of DH21 electrolyte is conducive to decreasing ion concentration gradient at the Zn deposition interface and preventing side reactions, which are believed to help the Zn deposition interface keep stable [22, 23]. The fitted distribution of relaxation times (DRT) data supported this conclusion (Fig. S5). We note that pDOL, with 2:1 ratio to H_2_O (DH21), has both excellent electrochemical stability window and desolvation capability. Therefore, the following experimental research is carried out on the electrolyte system under this ratio. Measurements of the contact angle between DH21 electrolyte and Zn foil also indicate that it contributes to the uniform distribution of Zn^2+^ flux at the electrode interface and the rapid transport of Zn^2+^ (Fig. S6). Compared with the aqueous electrolyte, DH21 electrolyte has a higher corrosion voltage (15.028 vs. 11.515 mV) and lower corrosion current density (1.55 vs. 5.87 mA cm^−2^, Fig. 2c).

X-ray absorption near-edge structure (XANES) optical photographs of Zn foil before and after the 20 days immersion test show that the Zn foil surface is heavily eroded in aqueous electrolyte, while the Zn foil surface remains smooth and clean in DH21 electrolyte (Fig. S7a, b). X-ray diffraction (XRD) results (Fig. S7c) further demonstrate that DH21 electrolytes can effectively inhibit Zn metal corrosion, which is very important for the long-term storage of Zn metal batteries. In the resting test, new peaks can be observed in the DRT for the Zn metal in aqueous electrolyte after 2 days of rest (Fig. S8a). The continuous accumulation with loose porous side products is then probed by XRD and scanning electron microscopy (SEM) after resting for 20 days (Fig. S8b), leading to a sharp impedance increase. In contrast, in DH21 electrolyte, the cell maintains a stable and side-reactions-free interphase, even after 21 days of rest (Fig. S8c). A dense and uniform morphology can be readily obtained when soaked in DH21 electrolyte for 20 days (Fig. S8d). In addition, Fig. S9 shows that the nucleation overpotential in DH21 electrolyte is higher than that in the aqueous electrolyte (7.9 mV). This higher nucleation overpotential is conducive to forming smaller and denser sites and promotes the uniform deposition of Zn^2+^ on the electrode surface. With the help of this 3D controlled diffusion in DH21 electrolyte, the Zn electrode is difficult to grow dendrites, which will effectively extend the cycle life (Fig. S10). Broad temperature and safety performance are very important for Zn metal batteries. Adding pDOL can destroy the hydrogen bond between H_2_O molecules and improve the low-temperature performance of the electrolyte. To study the low-temperature resistance of different electrolytes, as shown in the freezing experiment in Fig. 2d, aqueous, and DH12 electrolytes begin to freeze at − 20 °C, while DH11 and DH21 electrolytes have better anti-freezing property. The freezing points of aqueous, DH12, DH11, and DH21 electrolytes are − 10.7, − 16.8, − 21.8, and − 34.9 °C, respectively (Fig. S11). The ether-based solvent DOL is flammable, and it is worth noting that the results of the electronic ignition experiment simulating the electrical spark during the battery short circuit show that the separator soaked by the reconstructed polymerized DH21 electrolyte cannot be ignited by the electric spark (Fig. 2e). This is because the DOL monocase in this electrolyte has been catalyzed to polymerize into long-chain molecules by Zn salts (Zn(TFSI)_2_), and there are also different proportions of H_2_O in electrolyte. As a result, the DH21 electrolyte is no longer flammable, ensuring battery device safety in subsequent applications.

In order to determine the solvation structure of Zn^2+^, the local structures of Zn solvation shells in both electrolytes were detected by X-ray absorption fine structure (XAFS) spectroscopy analysis. Both electrolytes exhibit similar X-ray absorption near-edge structure (XANES) spectra, demonstrating that Zn^2+^ maintains a similar Zn valence state in both electrolytes (Fig. 2f). However, the Fourier transform of the EXAFS spectra concerning the DH21 electrolyte at the peak positions of 1.13 and 1.65 Å is significantly inconsistent with that of the aqueous electrolyte at the peak positions of 1.19 and 1.75 Å (Fig. 2g), indicating that the coordination structure of Zn^2+^ is significantly different. The shortening of the Zn–O bond length suggests that the introduction of pDOL causes partial H_2_O molecules in the solvated sheath of Zn^2+^ replaced by pDOL, forming a more anion-involved solvated structure. The change of solvation sheath regulates electrolyte stability, resulting in the formation of SEI film with different compositions on the surface of the Zn metal anode during the redox process. The SEI layer allows the transfer of Zn^2+^ while hindering the penetration of H_2_O, thus inhibiting the HER and improving the deposition efficiency of Zn^2+^ and the CE of the Zn metal batteries. The wavelet transform of EXAFS can analyze the different local structures of Zn^2+^, and the red region indicates the area where the peaks of R space and k space coincide. In aqueous electrolyte, it represents the Zn–O (O from H_2_O) coordination (Fig. 2h, i) and belongs to the highly dynamic and disordered solvation structure of H_2_O molecules mentioned earlier around Zn^2+^, making H_2_O molecules more prone to occur oxidation or reduction reaction, which quickly leads to the decomposition of electrolyte and formation of by-products [24]. By contrast, DH21 electrolyte has a more concentrated red region, indicating a more uniform and anion-involved solvation structure is conducive to improved solvent stability, which is also well reflected in the linear scan voltammetry (LSV) test in Fig. 2a.

### Evaluation of Interphase and Zn Electrodeposition of Zn Metal Anode

**Fig. 3:**
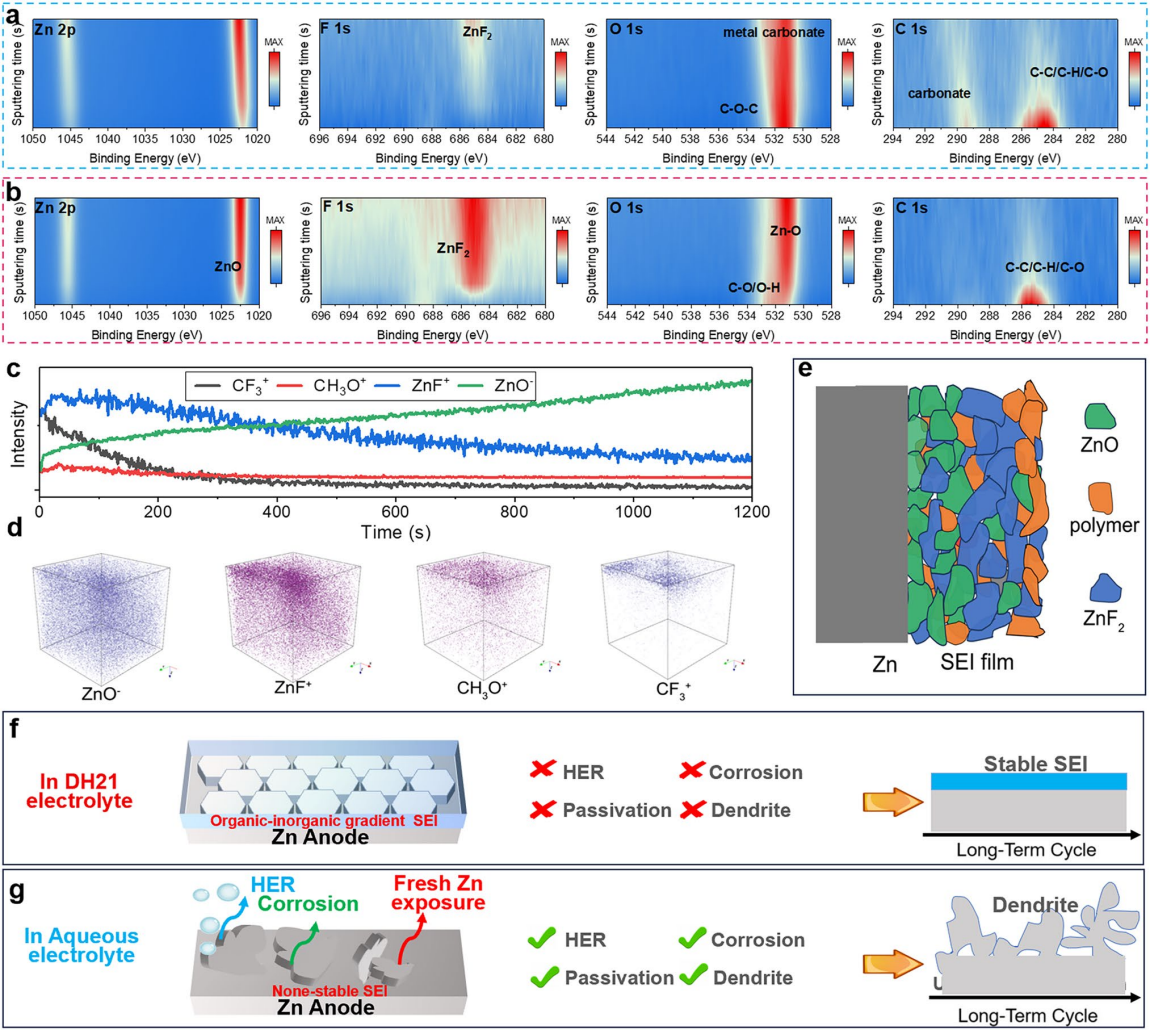
X-ray photoelectron spectroscopy (XPS) of Zn 2*p*, F 1*s*, O 1*s*, and C 1*s* of the Zn anode after cycle in **a** aqueous electrolyte and **b** DH21 electrolyte, which are displayed in rows, with corresponding durations of Ar^+^ sputtering in columns. **c** Time-of-flight secondary-ion mass spectrometry (TOF–SIMS) analysis of the SEI on Zn anode after 10 cycles in DH21 electrolyte, and **d** TOF–SIMS 3D render images of corresponding Zn electrode surface. **e** Diagram of SEI composition on Zn after 10 cycles in DH21 electrolyte. Schematic illustration of dendrites and side reaction evolution of Zn metal anodes in **f** DH21 electrolyte and **g** aqueous electrolyte

The elemental mapping images demonstrate the uniform elemental distribution of O, F, and Zn at the interphase. Focused ion beam-scanning electron microscope (FIB-SEM) image clearly displays a homogeneous SEI with a thickness of ∼3.6 μm on the Zn anode (Fig. S12). Nanoscale deep sputtering X-ray photoelectron spectroscopy (XPS) was applied to characterize the specific components of the formed SEI (Fig. 3a–d). The F 1*s* spectra show that the SEI layer of the Zn metal anode in both electrolytes after Ar^+^ etching is ZnF_2_ (685.5 eV), indicating the existence of a ZnF_2_-rich SEI layer [25, 26]. The O 1*s* spectra show that aqueous electrolyte on the surface of the Zn metal anode is dominated by carbonate and hydroxide peaks, while more ZnO in the DH21 electrolyte. C 1*s* spectra show that in the aqueous electrolyte, the electrode surface is occupied by more carbonate, while in DH21 electrolyte, with the increase in Ar^+^ etching time, the Zn electrode surface is basically free of carbonate produced by electrode corrosion, which indicates that DH21 electrolyte can inhibit the active H_2_O corrosion and related side reactions on the surface. Ga^+^ beam was used to further characterize the spatial distribution of SEI layer components in DH21 electrolyte by TOF–SIMS. In both positive and negative scanning modes, the depth profile of the SEI layer shows fluorine-rich characteristic. After etching more than 400 s, the fluorine content is still very high. With the increase in etching time, ZnO component in SEI increases, forming ZnO, ZnF_2_ compound, and a bidirectional gradient distribution of SEI (Fig. 3c) [27, 28]. The generated fluorine-rich organic–inorganic gradient SEI layer is conducive to the reversible cycle of Zn metal batteries. The spatial distribution of SEI components is shown in 3D scanning map with lateral resolution of 100 × 100 μm^2^. As shown in Fig. 3d, it is found that ZnO and ZnF_2_ present a gradient distribution, and CF_3_^+^ is only distributed in the surface layer, which belongs to the composition of the electrolyte. The mapping results also show that organic phases are also distributed in SEI. It is inferred that an electrolyte SEI structure is formed in DH21 electrolyte, as shown in Fig. 3e. ZnO and ZnF_2_ are distributed in gradient in the SEI, and the ZnO content is higher in the inner layer, which may be caused by the reduction process of pDOL on the Zn metal surface. At the same time, ZnF_2_ comes from the decomposition of electrolyte anion.

Decomposition products of solvents, solvated cations, and anions are the main components of the SEI dense layer. Before any interphase chemistry at the electrode occurs, a double-layer structure is formed at the electrode/electrolyte interface by the self-binding of solvent molecules, anions/cations, and electrode surface potentials, and this structure determines the phase interface chemistry of the battery. In DH21 electrolyte, when the surface of the Zn electrode is negatively charged, it will repel the anions in the inner layer, resulting in the formation of a thin and dense inorganic SEI layer dominated by ZnF_2_, ZnO, and inorganic elements. This dense layer has excellent mechanical property and the ability to conduct Zn^2+^ and isolate electrons, which has a significant effect on inhibiting the formation of dendrites and cutting off the direct contact between the electrolyte and Zn electrode to prevent HER and the occurrence of side reactions. After the formation of the inner SEI layer, the exterior is mainly accompanied by the irreversible reductive decomposition of the pDOL molecules in the electrolyte, which can form an organic SEI layer with good flexibility and compatibility with the electrolyte, and its presence makes the SEI layer smooth in general and effectively prevents the cracking of the SEI (Fig. 3f). Whereas ZnO is also formed at the Zn electrode/electrolyte interface in the aqueous electrolyte, the concurrent HER and passivation reactions lead to the formation of porous alkali sulfonates, which have poor Zn^2+^ transport capacity and are ineffective in preventing the subsequent Zn dendrite growth and side reactions, and ultimately the electrode still fails (Fig. 3g). This finding further solidifies the specific reversibility of Zn in the presence of DH21 electrolyte.

**Fig. 4:**
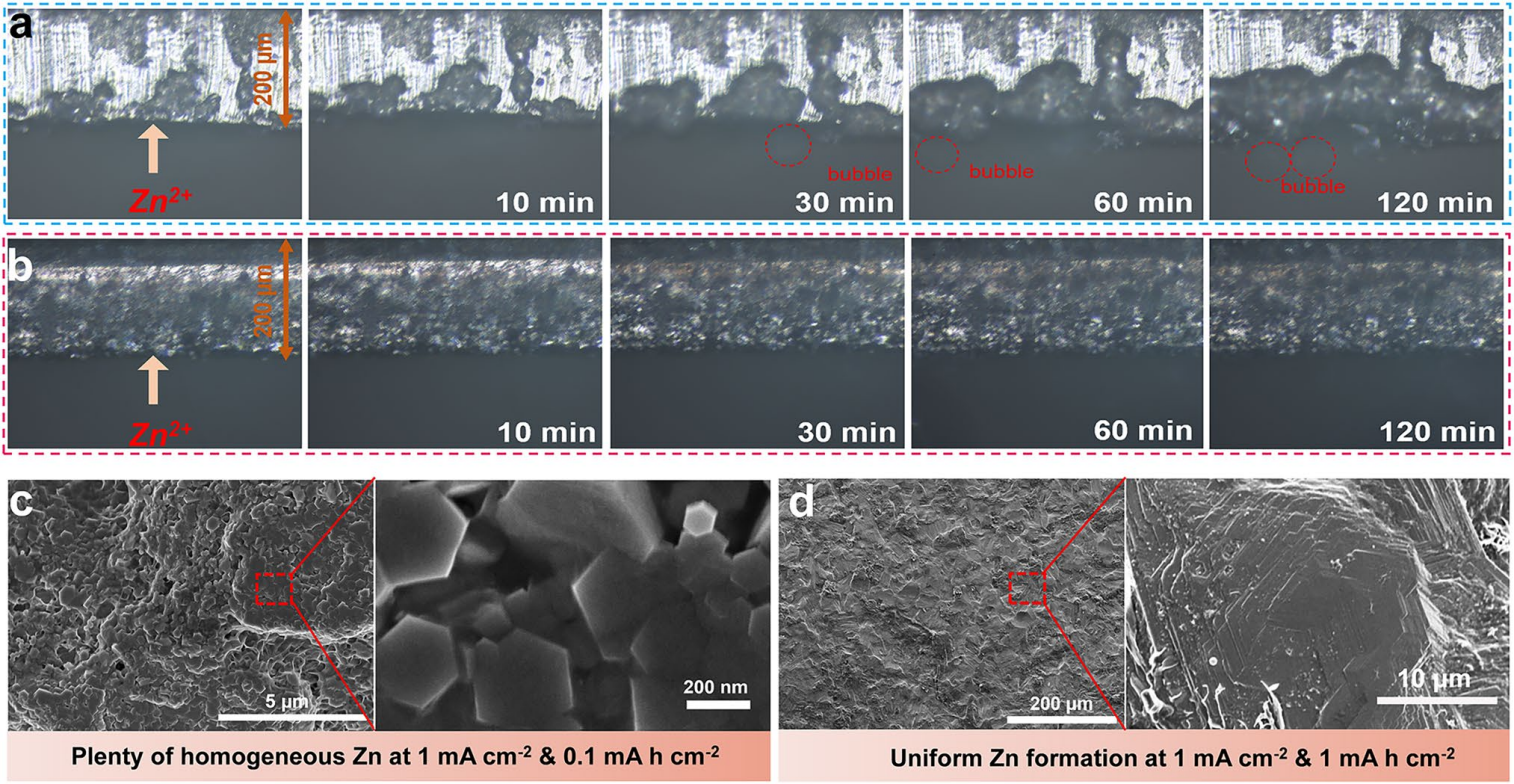
In situ optical microscope images of the Zn plating behavior in **a** aqueous electrolyte and **b** DH21 electrolyte. **c**, **d** SEM images of different Zn deposition capacities in DH21 electrolyte

The deposition behavior of Zn metal is crucial for maintaining the long-cycle stability of the electrode. There is a close and complex relationship between the deposition of metal anode and SEI [29]. Therefore, it is a challenging and important task to effectively regulate the composition of the SEI layer to guide good Zn deposition. Uniform and dense distribution of nuclei can inhibit irregular external growth and improve the densification of the electrode, and sufficient nuclei can inhibit dendrite growth and reduce porosity of the Zn metal electrode, stabilizing the Zn metal anode. Directing Zn nucleation in a way that modulates the SEI composition and structure may be an ideal strategy for constructing flat electrodes. Therefore, in this work, we further elucidate the intrinsic connection between gradient SEI formed by ZnO, ZnF_2_, and organic components and metallic Zn deposition. The real-time dynamic behavior of Zn plating at a current density of 2.0 mA cm^−2^ was observed by in situ optical microscopy. In the aqueous electrolyte system, Zn shows a non-uniform deposition pattern dominated by the "tip effect." Due to the unstoppable side reactions in the aqueous electrolyte system, bubbles generates by HER are observed, and dendrites becomes visible after 30 min of deposition, which most likely results in reduced reversibility and short circuit of the battery under operating conditions (Fig. 4a). In DH21 electrolyte, uniform deposition of Zn along the electrode/electrolyte interface is observed, confirming the positive effect of DH21 electrolyte in stimulating nucleation sites and inducing uniform deposition (Fig. 4b). SEM was used to observe the morphology of Zn deposition in the plated layers at 0.1 mA cm^−2^ and 1.0 mAh cm^−2^. The Zn anode in the aqueous electrolyte shows a random and irregular orientation (Fig. S13). In contrast, for Zn circulating in the DH21 electrolyte (Fig. 4c), a high degree of orientation can be clearly recognized, and the Zn deposition morphology is hexagonal. As the deposition capacity increases, we find that the crystal size of metallic Zn further increases and grows into dense and similarly large-sized Zn (Fig. 4d). We have demonstrated that in the DH21 electrolyte, it is advantageous to obtain dense and uniform SEI protective layer with better adhesion, which has zinc-friendly ZnO at the bottom of the SEI in contact with Zn and highly zinc-ion-conducting ZnF_2_ in the middle, while the polymer component runs through it in the form of binder, improving the mechanical strength of the SEI. Compared to Zn metal, the ZnO layer has a strong adsorption capacity for Zn^2+^ (Fig. S14), and Zn^2+^ is adsorbed on the uniformly distributed ZnO surface in the SEI, which promotes favorable nucleation and uniform deposition of Zn^2+^ [30]. The high binding of ZnO to Zn^2+^ promotes uniform trapping of Zn^2+^, leading to the formation of a large number of nucleation sites. Subsequently, Zn^2+^ is reduced and deposited on the surface of the Zn electrode under an applied electric field, which ensure high interfacial stability during Zn deposition and ultimately leads to dendrite-free Zn deposits underneath the SEI layer during the plating process. In addition, ZnF_2_ has a high dielectric constant and high Zn^2+^ conductivity, and the organic component in it improves the mechanical strength, which ensures fast and uniform Zn^2+^ transport and structural stability of the SEI layer during charging and discharging. The SEM images show that in DH21 electrolyte, as the deposition capacity increases, the nuclei grow into a large number of small-sized, dense and uniform hexagonal prism-shaped single-crystal Zn particles with no dendritic crystals appearing. As cumulative deposition proceeds eventually flat and large-sized Zn are formed. In the aqueous electrolyte, due to the lack of a stable and ZnO-rich SEI layer, the initial nucleation of Zn deposition is not uniform, and the nucleation density is low, and as the deposition proceeds, a chaotic and disordered plating layer appears, and ultimately a large number of dendrites are formed and the cell is short circuited. In conclusion, the organic–inorganic hybrid SEI formed in DH21 electrolyte improves mechanical protection and constructs an electrodynamic barrier, avoiding unstable interfaces, and more importantly, ZnO can guide the formation of favorable Zn nuclei, which promotes uniform Zn deposition. Ultimately, the practical application of advanced Zn metal batteries is facilitated.

### Compatibility with Zn Metal Anode

**Fig. 5:**
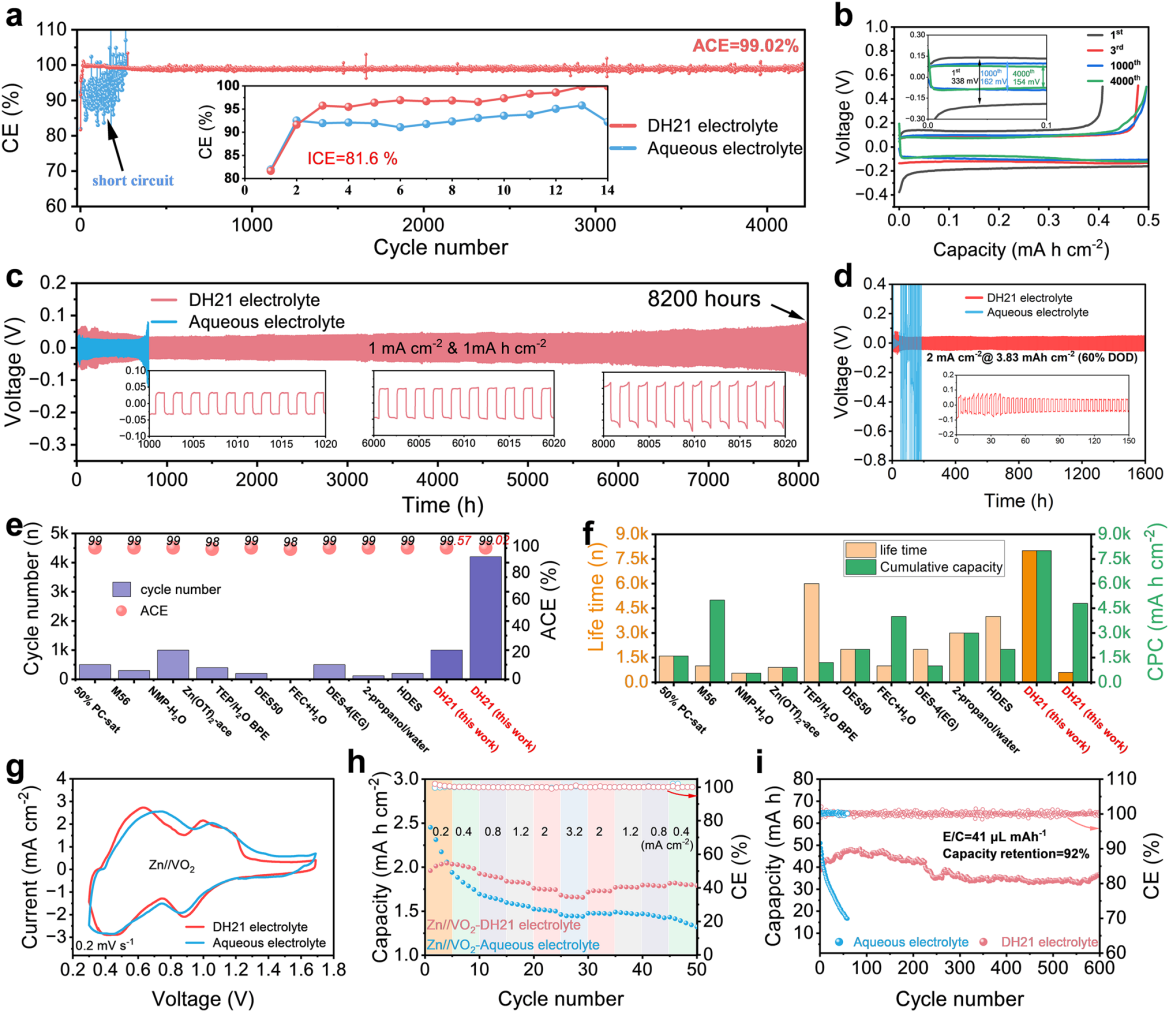
**a** Coulombic efficiencies of Zn//Cu asymmetric cells with both electrolytes at 1 mA cm^−2^ with 0.5 mAh cm^−2^ (inset with magnified views of selected 14 cycles). **b** Zn plating/stripping profiles on Cu foil in DH21 electrolyte. **c** Long-term galvanostatic Zn plating/stripping in Zn//Zn symmetric cells at 1 mA cm^−2^ with a specific capacity of 1 mAh cm^−2^. **d** Long-term galvanostatic Zn plating/stripping in Zn//Zn symmetric cells at 60% DOD. **e**, **f** Cycle number, ACE, lifetime, and CPC comparison of the cells using DH21 electrolyte with others reported literature. **g** CVs of the Zn//VO_2_ pouch batteries at the scan rate of 0.2 mV s^−1^. **h** Rate and **i** cycling performance of the Zn//VO_2_ pouch batteries in both electrolytes

The CE of Zn plating/stripping on Cu foil in both electrolytes was studied. As shown in Fig. 5a, the Zn//Cu asymmetric cells in aqueous electrolyte short circuit quickly, with continuous and significant fluctuations. This phenomenon indicates that the electrode/electrolyte interface of aqueous electrolyte is unstable, accompanied by dendrite growth and overcharging caused by the accumulation of "dead Zn" and by-products. The initial Coulomb efficiency (ICE) of the cell in DH21 electrolyte is 81.6%, rapidly increasing to 99% with the stable SEI produced. In the subsequent 4200 cycles, the cell has no significant fluctuations and provides a satisfactory average Coulomb efficiency (ACE = 99.02%). The voltage curves of the Zn//Cu asymmetric cells for different cycles in both electrolytes also indicate that the Zn metal anode has a more stable performance in DH21 electrolyte (Figs. 5b and S15). The results indicate that the introduction of pDOL in the electrolyte inhibits side reactions and uniform Zn deposits at the electrode interface. At higher current density and deposition capacity (3.82 mA cm^−2^, 2.10 mAh cm^−2^), the plating/stripping efficiency of Zn^2+^ in DH21 electrolyte is also significantly improved compared with aqueous electrolyte (ACE = 99.57%); interestingly, the cycle life of the Zn metal anode is also extended considerably (Fig. S16). Then, the electrochemical properties of Zn metal anodes in both electrolytes were measured by the symmetrical cells with constant current cycle measurement. Furthermore, as shown in Fig. 5c, with DH21 electrolyte, the cell can achieve a remarkably long cyclic lifespan of 8200 h, revealing the high reversibility of Zn plating/stripping. In contrast, the Zn//Zn symmetric cell with aqueous electrolyte short circuits after only 750 h. The excellent rate performance of the cell with DH21 electrolyte is also reflected in Fig. S17. At a higher current density of 8 mA cm^−2^ with 1 mAh cm^−2^, the cell can be stably cycled in DH21 electrolyte for more than 500 h, achieving a lifetime extension of nearly 40 times compared to aqueous electrolyte (Fig. S18). The optical photographs and SEM images of the Zn metal anodes after cycling in the DH21 electrolyte have a flatter surface, indicating no significant Zn dendrite production (Fig. S19). The micro-FTIR and XRD results of the electrode plates after cycling also show that the Zn surface in DH21 electrolyte is not covered by by-products (Figs. S20 and S21). Achieving stability at high DOD for Zn metal anode will improve the energy density of rechargeable Zn metal batteries in practical applications, but it remains a huge challenge. Due to the regulatory effect of pDOL in the electrolyte on Zn^2+^ flow at the interface of the Zn electrode, the Zn metal anode can cycle stably for more than 2500 h at high 60% DOD, 30 times longer life than in aqueous electrolyte (Fig. 5d). The slightly higher overpotential of the Zn//Zn symmetric cell in DH21 electrolyte may be related to its relatively low ionic conductivity and the regulation of Zn^2+^ transport by forming SEI, which will be analyzed in detail below. Although the overpotential is slightly higher, the cycle life and stability of the Zn metal anode are greatly improved. As mentioned above, DSC results show that DH21 electrolyte has a low freezing point of − 34.9 °C, so its electrochemical properties at low temperatures were investigated. Impressively, even at a low temperature of − 10 °C, the Zn//Zn symmetric cell assembled with DH21 electrolyte demonstrates a stable cycle of 1000 h (Fig. S22a). In Fig. S22b, the performances of Zn//Zn symmetric cells with both electrolytes at different temperatures were compared. Zn//Zn symmetric cell does not show significant low-temperature advantage with aqueous electrolyte, and the polarization increases significantly at − 15 °C. When the temperature drops to − 20 °C, a sharply fluctuating voltage curve and rapidly rising overpotential are observed. Finally, the battery fails due to excessive polarization. In contrast, Zn//Zn symmetric cell with DH21 electrolyte can still maintain a stable cycle at − 25 °C. This shows that it has good stability and good low-temperature applicability, which greatly expands the application scenario of the electrolyte. Finally, we compared the properties of DH21 electrolytes with those reported co-solvent electrolytes in recent years and found that DH21 electrolyte exhibits higher CE, lifetime, and cumulative deposition capacity (Fig. 5e, f and Tables S2–S3) [10, 18, 31–38].

Vanadium-based oxides cathodes have drawn extensive attention due to plenty of merits originating from multivalence characteristics. Especially, VO_2_ with a vast tunnel structure (4.98 Å × 3.28 Å) can store the divalent Zn^2+^ charge carriers without large lattice shearing. Importantly, it can provide a high theoretical capacity of 322.9 mAh g^−1^ [39]. To further demonstrate the practical advantages of DH21 electrolyte, we assembled a lean liquid practical Zn//VO_2_ full cells. Figure 5g shows the cyclic voltammetry (CV) curves of the Zn//VO_2_ full cells at a scan rate of 0.2 mV s^−1^. Zn//VO_2_ full cells show similar reversible redox behavior in both electrolytes, consistent with the previous findings [39]. The rate capability of VO_2_ cathode in both electrolytes was tested (Figs. 5h and S23). We evaluated the stability of the full cell in both electrolytes and compared the voltage attenuation during the standing process (Fig. S24). It was found that the initial discharge capacity of the cells is similar in both electrolytes. After resting 12 h for self-discharge test, the voltage of the coin cell with aqueous electrolyte drops from 1.60 to 1.245 V, but for DH21 electrolyte, the voltage drops from 1.60 to 1.286 V. After three charge–discharge cycles, the attenuation of discharge capacity in aqueous electrolyte is more serious than that in DH21 electrolyte, which indicates that DH21 electrolyte improves the performance of the full cell. Fortunately, the rate capability and stability of VO_2_ cathode in DH21 electrolyte are effectively improved. As the current density increases, the battery in DH21 electrolyte exhibits a higher capacity. In the previous reports, the excessive use of electrolytes covers the consumption problem caused by side reactions and makes the electrochemical properties evaluation different from the practical scenario. As shown in Fig. 5i, the capacity drops sharply after only 50 cycles in aqueous electrolyte, and then, the battery fails. The degradation of the control battery is mainly due to the growth of Zn dendrites and the continuous decomposition and consumption of electrolytes. In contrast, even with a low N/P ratio (13.9) and E/C ratio (41 mL (Ah)^−1^), the cell utilizing DH21 electrolyte still presents impressive durability over 600 cycles with a capacity retention ratio of 92% (Fig. 5i). After cycling, the optical photographs of the pouch battery show an obvious bulge problem in the aqueous electrolyte, but no bulge in the DH21 electrolyte (Fig. S25). The above experimental results confirm the success of realizing high-performance Zn metal batteries in solvent reconfiguration engineering enabled Zn(TFSI)_2_-mediated ring-opening polymerization.

## Conclusions

In summary, the pDOL-enhanced electrolyte has good antioxidant stability and non-combustible enabled by Zn(TFSI)_2_-mediated ring-opening polymerization. The novel electrolyte not only reduces the active H_2_O, widens the electrochemical window of the electrolyte, but also improves low-temperature performance and assists in the formation of an organic–inorganic gradient SEI with rich organic constituent, ZnO and ZnF_2_. The side reaction induced by H_2_O in the DH21 electrolyte is effectively inhibited, the reversibility of Zn plating/stripping is greatly improved, and the formation of Zn dendrites is inhibited. Thus, it delivers high reversibility of dendrite-free Zn plating/stripping in DH21 electrolyte (4200 cycles with 99.02% ACE in Zn//Cu asymmetric cell; lifespan of 8200 h at 1 mA cm^−2^ and 2500 h at high DOD of 60% in Zn//Zn symmetric cells; 1000 h at low temperature of − 10 °C). More importantly, the Zn//VO_2_ pouch battery assembled in the lean liquid state (E/C = 41 mL (Ah)^−1^) has a capacity retention ratio of 92% after 600 cycles. This work provides a new idea for the practical application of Zn metal batteries.

## Supplementary Information

Below is the link to the electronic supplementary material.

Supplementary file 1 (DOCX 10197 KB)
