# Supplementary material for: Zn(TFSI)2-Mediated Ring-Opening Polymerization for Electrolyte Engineering Toward Stable Aqueous Zinc Metal Batteries
Source: Nanomicro Lett. 2025 Jan 28;17:120. doi: 10.1007/s40820-025-01649-9 (PMC11775372; doi:10.1007/s40820-025-01649-9)
Supplement: Supplementary file 1 — (DOCX 10197 KB) [file 40820_2025_1649_MOESM1_ESM.docx]

Supporting Information for

**Zn(TFSI)_2_ Mediated Ring-Opening Polymerization for Electrolyte Engineering towards Stable Aqueous Zinc Metal Batteries**

Zhenjie Liu^1‡^, Murong Xi^1‡^, Rui Sheng^1^, Yudai Huang^1^*, Juan Ding^1^, Zhouliang Tan^1^, Jiapei Li^2^, Wenjun Zhang^2^*, Yonggang Wang^3^*

^1^ State Key Laboratory of Chemistry and Utilization of Carbon Based Energy Resources, College of Chemistry, Xinjiang University, Urumqi 830017, P. R. China

^2^ Department of Materials Science and Engineering & Center of Super-Diamond and Advanced Films, City University of Hong Kong, 83 Tat Chee Avenue, Kowloon, Hong Kong SAR, P. R. China

^3^ Department of Chemistry and Shanghai Key Laboratory of Molecular Catalysis and Innovative Materials, Institute of New Energy, iChEM (Collaborative Innovation Center of Chemistry for Energy Materials), Fudan University, Shanghai 200433, P. R. China

^‡^ Zhenjie Liu and Murong Xi contributed equally to this work.

*Corresponding authors. E-mail: [huangyd@xju.edu.cn](mailto:huangyd@xju.edu.cn) (Yudai Huang); [apwjzh@cityu.edu.hk](mailto:apwjzh@cityu.edu.hk) (Wenjun Zhang); [ygwang@fudan.edu.cn](mailto:ygwang@fudan.edu.cn) (Yonggang Wang)

**S1 Experimental Section**

**S1.1 Chemicals and Materials**

Chemicals ((Zn(TFSI)_2_, >99%) and (1,3-Dioxolane (DOL), 99.5%, contains ~75 ppm BHT as inhibitor)) used in this study were purchased from Adamas-beta®, and were used as received without any further purification, unless otherwise specified.

**S1.2 Preparation of electrolyte**

A series of electrolyte of Zn(TFSI)_2_ with varying DOL/H_2_O ratios were prepared. Specifically, a certain amount of Zn(TFSI)_2_ was dissolved in pre-mixed DOL/H_2_O. After that, the mixture is placed in a constant temperature oven at 80 °C for 12 h and then left for 12 h to complete the ring-opening polymerization process.

***Preparation of VO_2_ cathodes*** The VO_2_ cathodes were prepared by mixing VO_2_ powder (99%, Adamas), PTFE, active carbon (AC), carbon nanotubes (CNTs) and SiO_2_ (10 nm) with a weight ratio of 7:1:1:0.5:0.5. Then, the prepared dry powder was pressed onto a stainless-steel mesh by roller press. After fibrillation at 100 °C for 10 h, the electrodes were cut into discs with diameters of 12 mm, and the area capacity of VO_2_ is approximately 2-4 mAh cm^-2^. The thickness of Zn foil tested for the pouch battery used is 50 μm (capacity is 29.27 mAh cm^-2^), and the average discharge capacity of the VO_2_ cathode is 2.1 mAh cm^-2^. Based on the above data, the N/P of the full cell is 13.9.

Zinc foil with a 0.1 and 0.01 mm thickness was purchased from *1688.com*. The Zn foil of 0.01 mm and copper foil roller composite were used as the anode for DOD testing (Zn@Cu). The area capacity of the Zn@Cu foil after rolling is 6.38 mAh cm^-2^, which is based on the maximum capacity of Zn stripping. Glass fiber separator (Olegeeino GF/A2916, cut into Φ16 mm) was used as the separator. For Zn//Zn symmetric cells, Zn foils were assembled into the 2032 coin-type cells with 80 μL electrolyte. Zn//Cu asymmetric cells were used to measure the Zn stripping/plating reversibility (i.e. Coulombic efficiency). Then the assembled cells stand for 12 h before electrochemical performance.

**S1.3 Characterization**

The phases of Zn anode before and cycled were characterized by X-ray diffraction (XRD, Bruker D8 Advance equipped with Cu Kα radiation, Germany) and the morphology was characterized by field-emission scanning electron microscopy (SEM, Hitachi FE-SEM S-4800, Japan). The Raman spectrum was measured by a Bruker Senterra-R200-L spectrometer (532 nm). The measurement of surface wettability of electrodes was performed by a T-JC2000C1 contact angle measuring system (POWER EACH, Shanghai, China). Spotlight 400 FT-IR Imaging System was used for the MIR test. TOF-SIMS analysis was characterized by TOF-SIMS 5 5-100 instrument (IONTOF GmbH). RX50M metallographic microscope (Sunny Optical, Ningbo, China) is used for optical magnification to observe the morphology of electrodes and to set up in-situ observation platform.

We applied the neutralizer during our XPS test. The Ar sputter gun was used for the etching with the ion energy set at 200 eV and the middle range current was selected. The sputtering rate was estimated to be ~0.01 nm s^-1^. The sample was directly moved from the Ar atmosphere to the XPS chamber with a vacuum transfer container to avoid exposure to the air. The etching procedure was carried out in a cycle of accumulated 0, 10, 30, 60, 100, and 200 s. Spectra were recorded of the sample surface before sputtering and between sputtering cycles.

X-ray absorption fine structure (XAFS) spectroscopy was carried out using the Rapid XAFS 2M (Anhui Absorption Spectroscopy Analysis Instrument Co., Ltd., China) by transmission mode at 20 kV and 40 mA. The obtained XAFS data was processed in Athena (version 0.9.26) for background, pre-edge line and post-edge line calibrations. Then Fourier transformed fitting was carried out in Artemis (version 0.9.26). For Wavelet Transform analysis, the χ(k) exported from Athena was imported into the Hama Fortran code.

**S1.4 Electrochemical Measurements**

Cyclic voltammetry (CV) and linear sweep voltammetry (LSV) curves were obtained by an electrochemical workstation (VMP-300 electrochemical workstation, France). The galvanostatic charge/discharge (GCD) measurements were performed by a battery test system (LAND Battery Test System (CT2001A) and NEWARE Battery Test System (MHW-300-5V12A80CH-380V-3C), China). The electrochemical impedance spectra (EIS) were measured in a frequency range from 10 mHz to 1 MHz with an AC voltage amplitude of 5 mV (ZAHNER-elektrik electrochemical workstation and VMP300 electrochemical workstation, Germany). The distribution of relaxation times (DRT) analysis was conducted by applying the Python GUI based pyDRT tools, which are developed by Ciucci’s group. The parameters for DRT are based on the Gaussian discretization method with a regularization parameter of 10^−3^ and a FWHM control of 0.5. Based on the DRT analysis, the equivalent circuit models were established, and parameters involved in the electrochemical processes such as the diffusion resistance (R_diffusion_) and the charge transfer resistance (R_ct_) were fitted. The values of activation energy for the charge transfer process were further calculated according to the Arrhenius equation as follows:

𝑅_ct_ = 𝑘e^−𝐸a/R𝑇^ (S1)

where R_ct_ represents the charge transfer resistance, k is the pre-exponential factor, R is the molar gas constant (8.314 J mol^−1^ K^−1^), T is the applied temperature in the EIS measurements, and Ea is the activation energy.

**S1.5 Calculation of the cumulative plating capacity**

Cumulative plating capacity = current density × total Zn plating time

**S1.6 Theoretical Simulations**

The electrostatic potential calculations (ESPs) of the DOL and pDOL were conducted by Gaussian (G09) program9 with Becke’s three-parameter hybrid method using the Lee-Yang-Parr correlation functional (B3LYP) at 6-311++G(d,p) level [S1]. Frequency analysis was performed to ensure the ground state of optimized molecules.

**S2 Supplementary Figures and Tables**


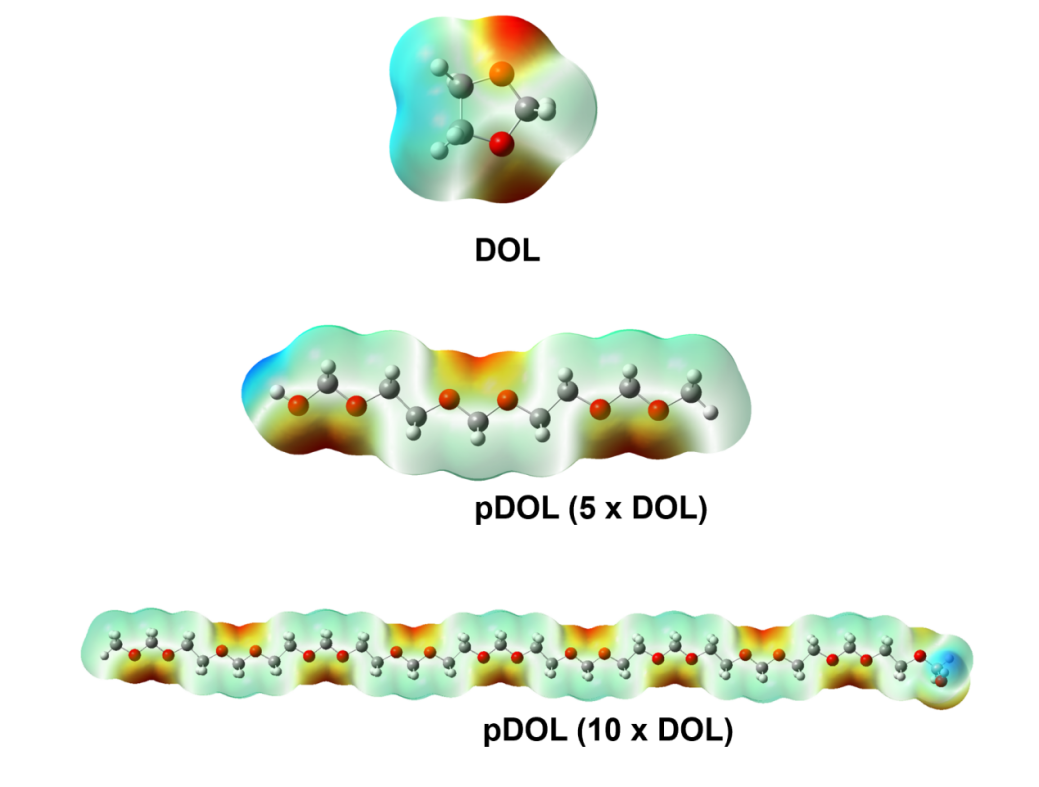


**Fig. S1** Electrostatic potential (ESP) diagram of DOL and pDOL


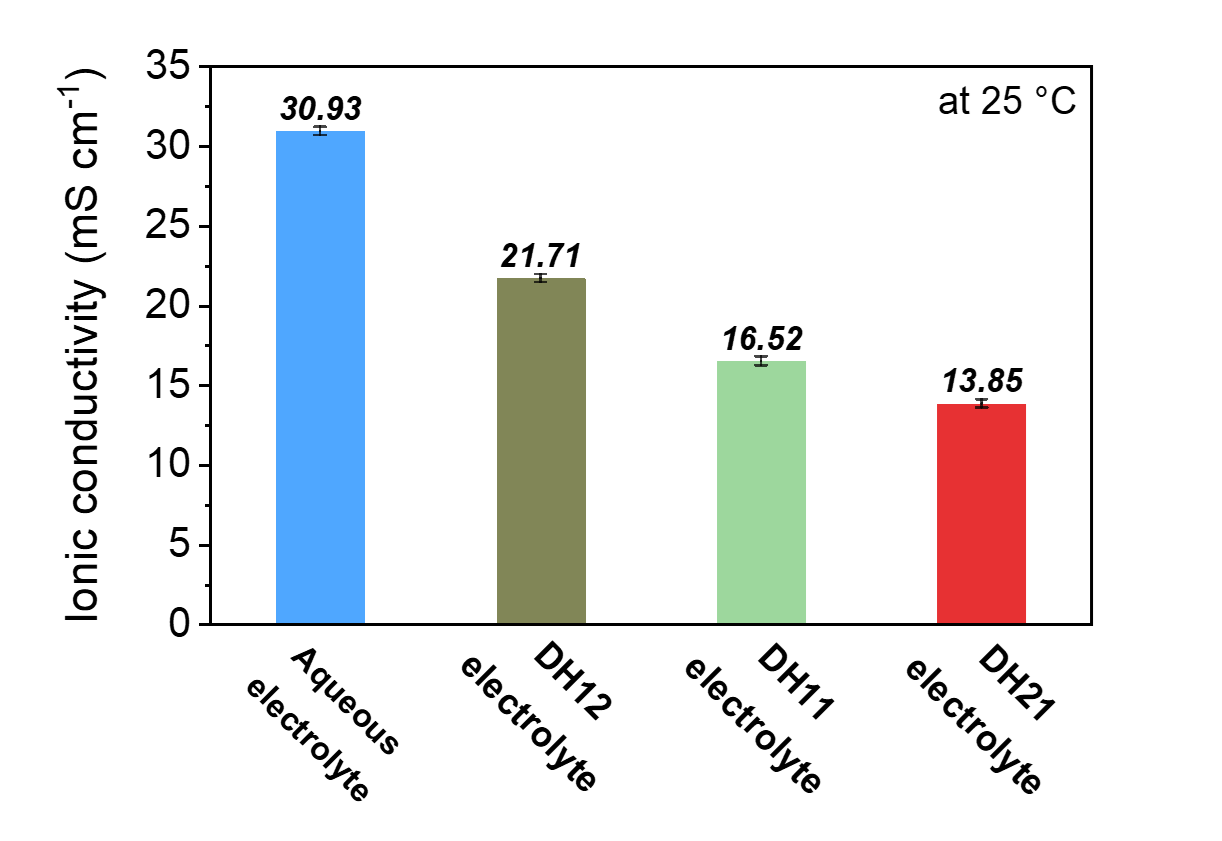


**Fig. S2** ionic conductivity of different electrolytes

The ionic conductivity of aqueous electrolyte (0.5M Zn(TFSI)_2_ in H_2_O) is 30.93 mS cm^-1^, and the ionic conductivity of DH21 electrolyte is 13.85 mS cm^-1^.


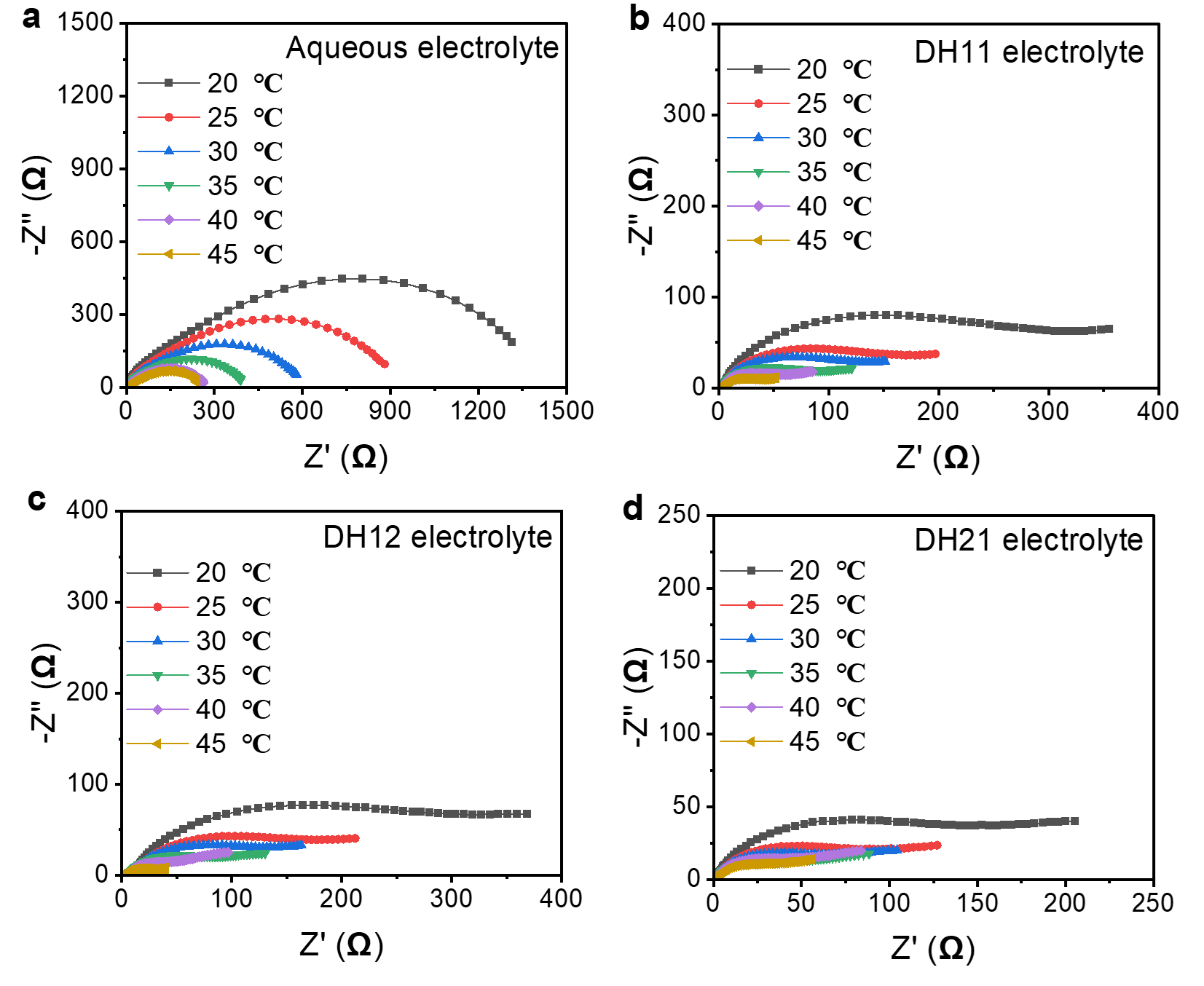


**Fig. S3** EIS of Zn anode in different electrolytes at different temperatures. (**a**) aqueous electrolyte, (**b**) DH11 electrolyte, (**c**) DH12 electrolyte, and (**d**) DH21 electrolyte

**
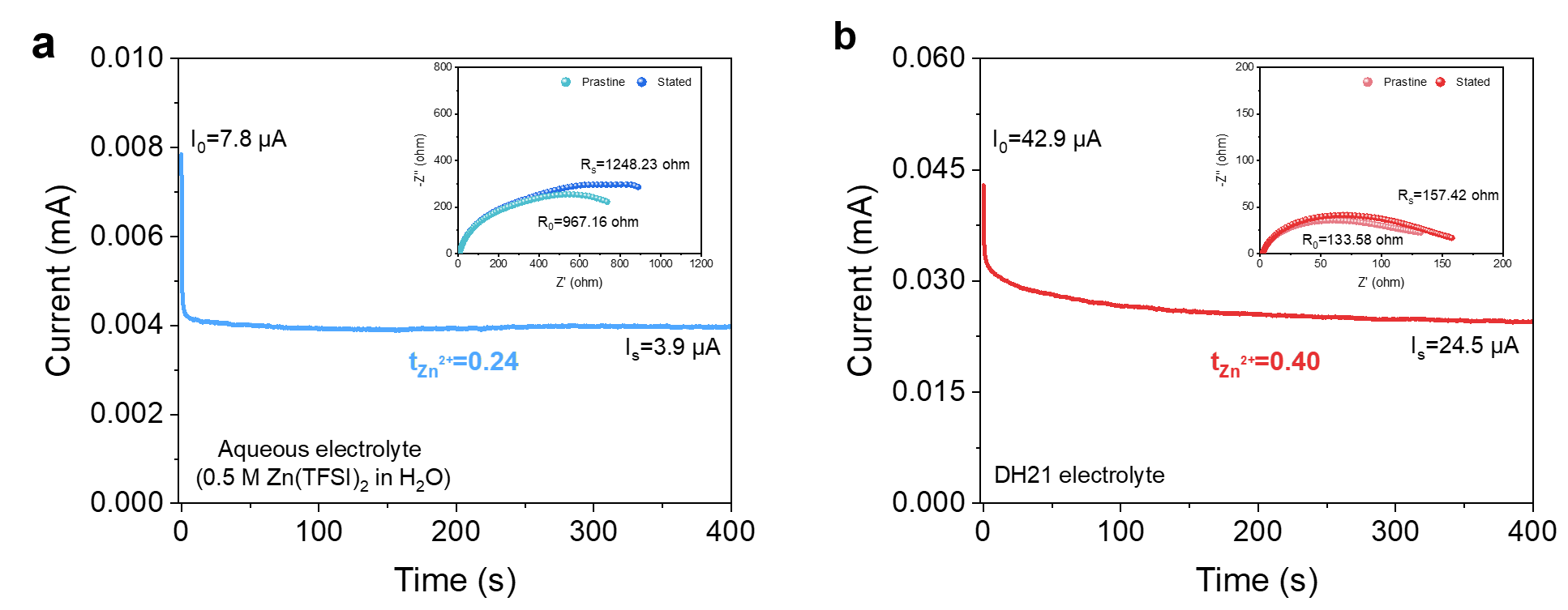
**

**Fig. S4** Chronoamperometry (CA) tests of Zn//Zn symmetric cells with (**a**) aqueous electrolyte and (**b**) DH21 electrolyte. Insets are EIS spectra of these symmetric cells before and after CA tests. Zn^2+^ transference number is calculated to be 0.24 for (**a**) aqueous electrolyte. and 0.40, for (**b**) DH21 electrolyte


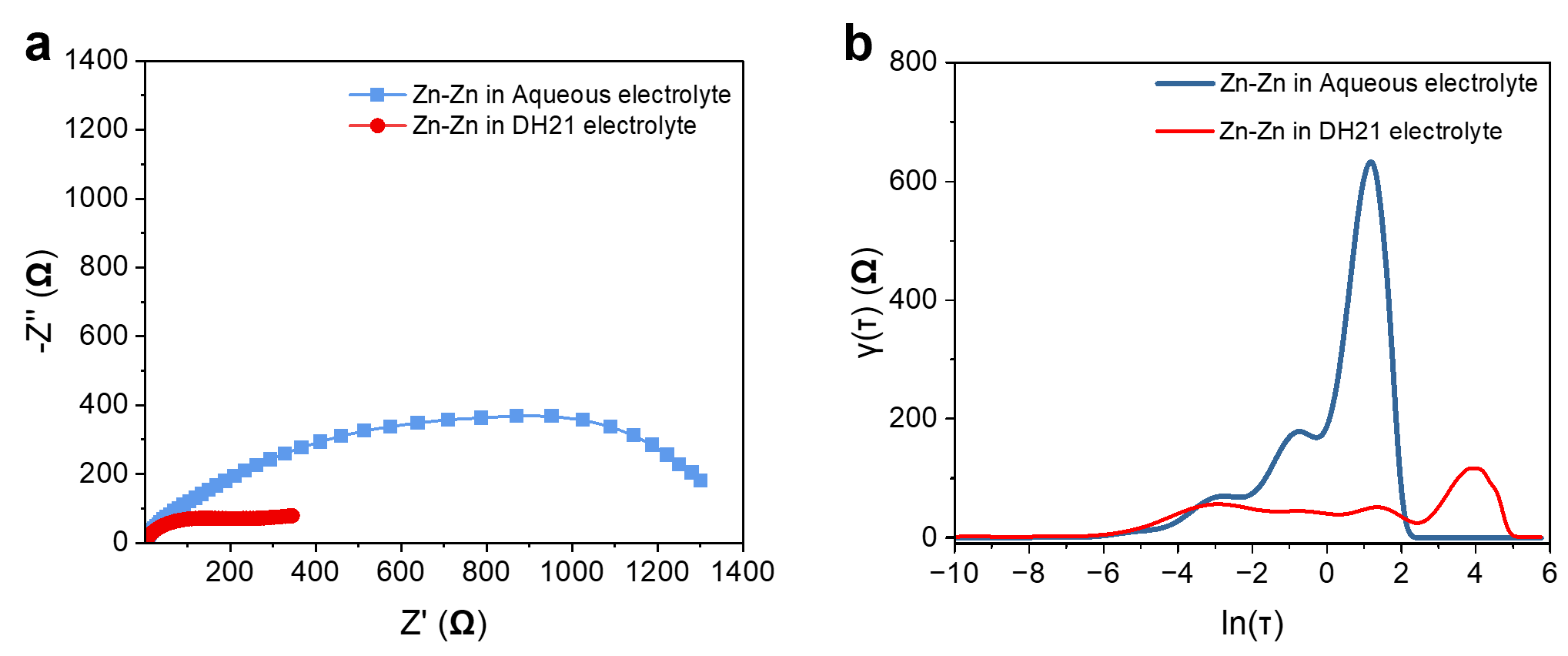


**Fig. S5** (**a**) EIS spectra of Zn//Zn symmetrical cells in different electrolytes and corresponding (**b**) analysis of the distribution of relaxation times (DRT) upon galvanostatic


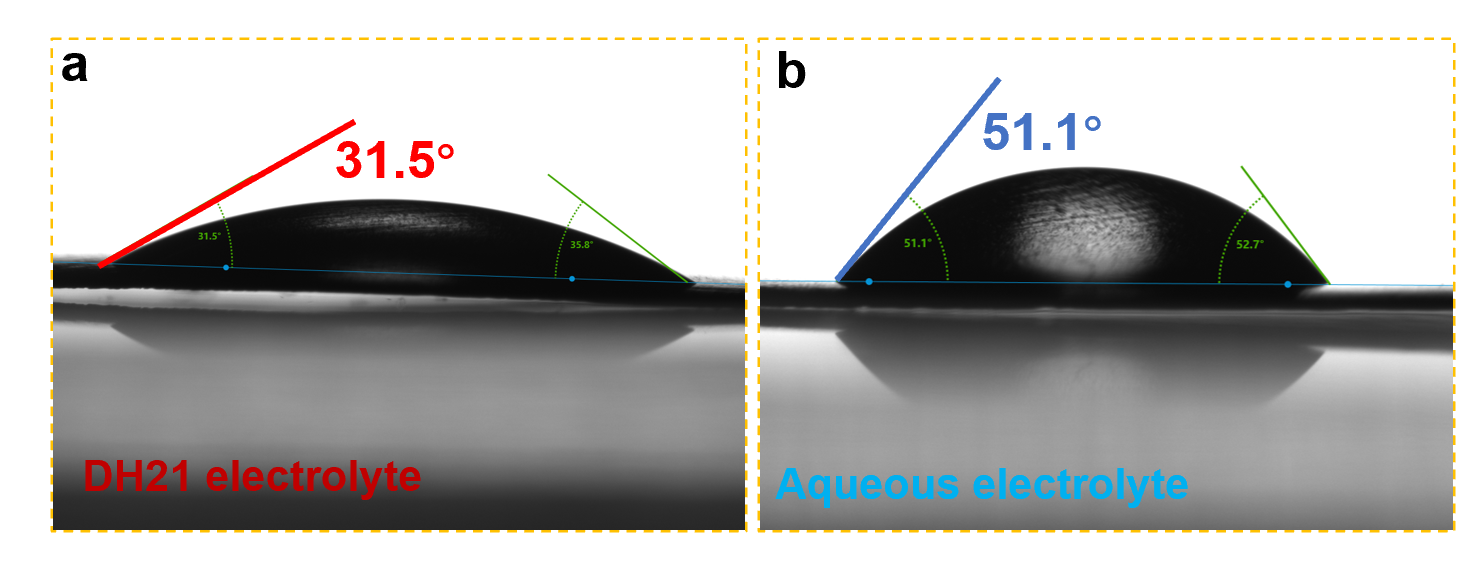


**Fig. S6** Contact angle of (**a**) DH21 electrolyte, and (**b**) aqueous electrolyte on Zn foil

The good contact between electrolyte and electrode interface enables the uniform distribution of Zn^2+^ on electrode interface. As shown in Fig. S6, the instantaneous contact angle of DH21 electrolyte captured by the contact angle test instrument on the electrode surface is only 35°, while the instantaneous contact angle of aqueous electrolyte on the Zn electrode surface is greater than 50°. The above results show that the good wettability of DH21 electrolyte can reduce the interfacial free energy between the Zn substrate and the electrolyte thermodynamically, which is conducive to the formation of uniform Zn nucleation site at the initial discharge to obtain uniform Zn deposition.


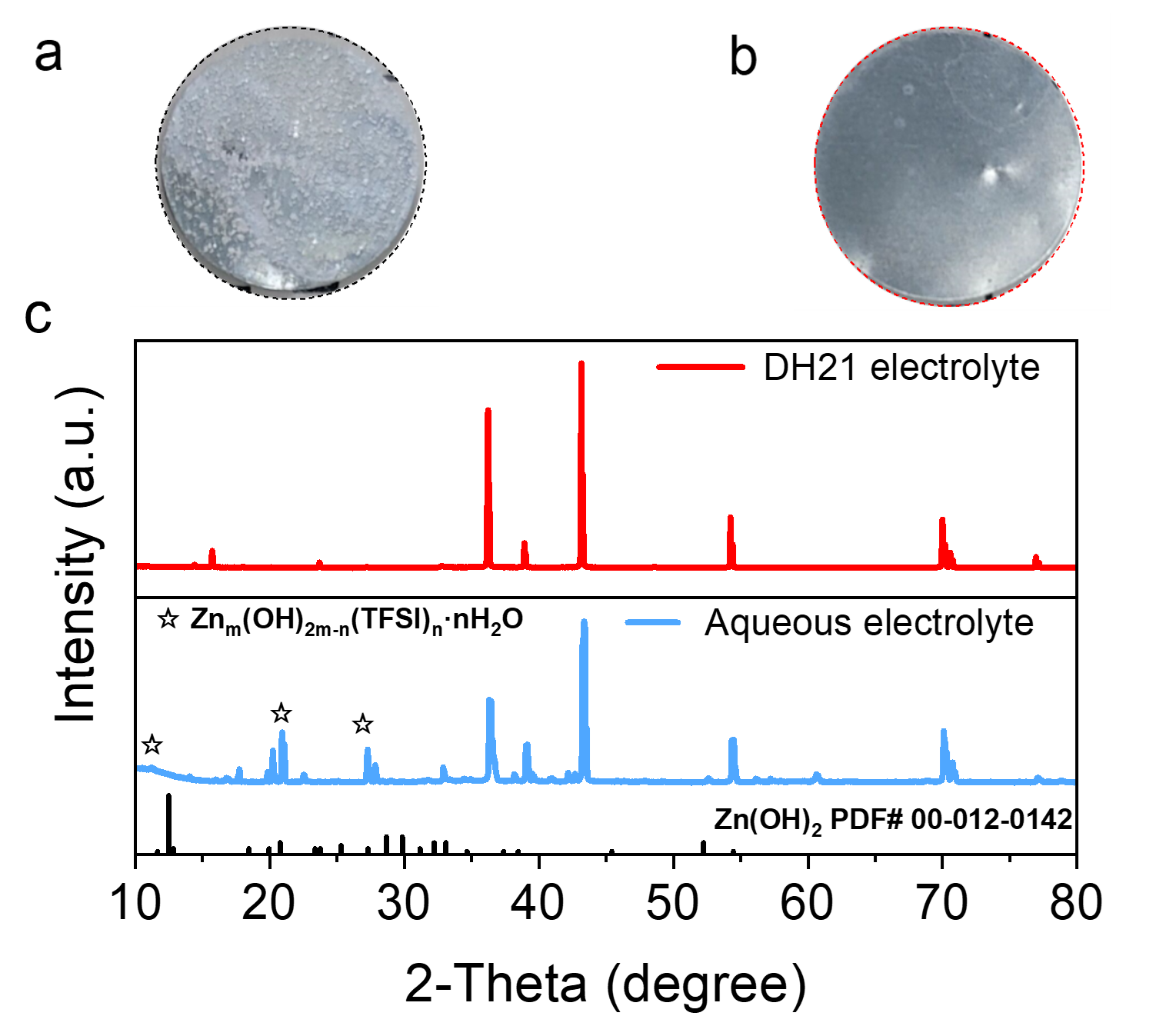


**Fig. S7** The optical photographs of Zn foil after 20 days in (**a**) Aqueous electrolyte, and (**b**) DH21 electrolyte. (**c**) XRD of the Zn anode soak in both electrolytes for 20 days


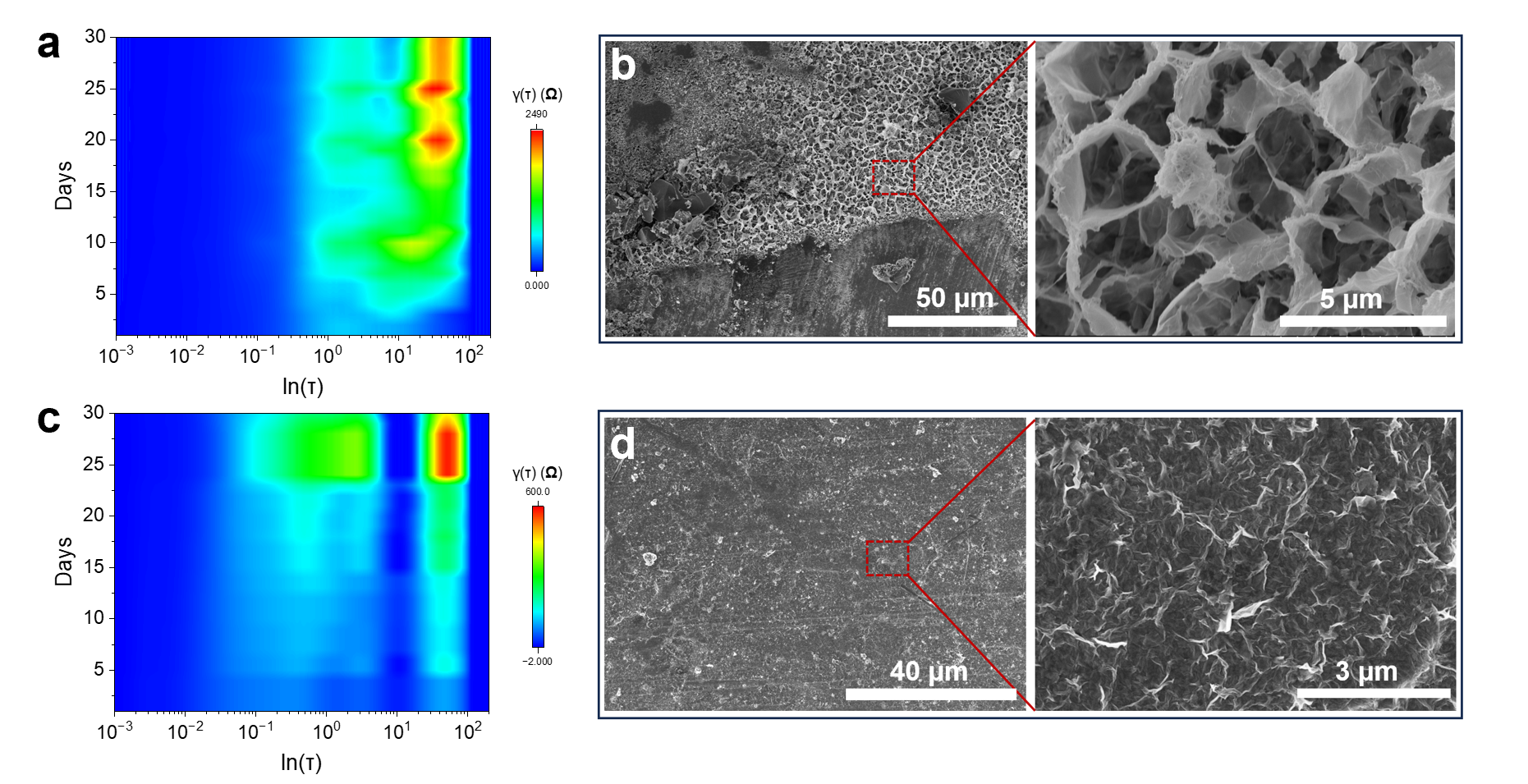


**Fig. S8** DRT analysis of Zn//Zn symmetric cells in (**a**) aqueous electrolyte, and (**c**) DH21 electrolyte by operando EIS evolution during standing. Micrographs of Zn foil after 20 days in (**b**) aqueous electrolyte, and (**d**) DH21 electrolyte


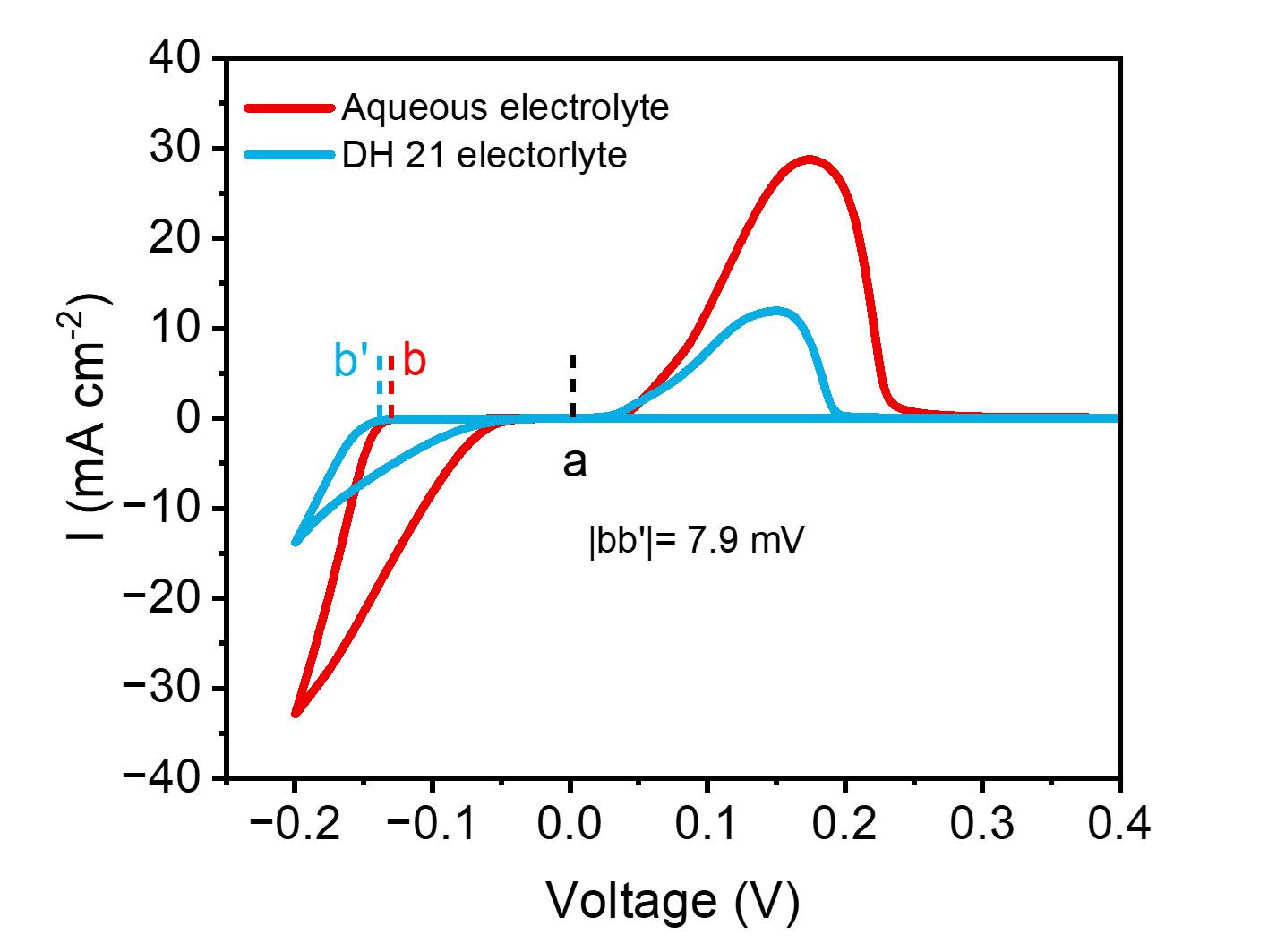


**Fig. S9** CV curves of Zn on Cu in both electrolytes

The 7.9 mV nucleation overpotential in DH21 electrolyte higher than that in aqueous electrolyte indicates that Zn electrode has smaller and denser nucleus formation in DH21 electrolyte, which promotes the uniform deposition of Zn.


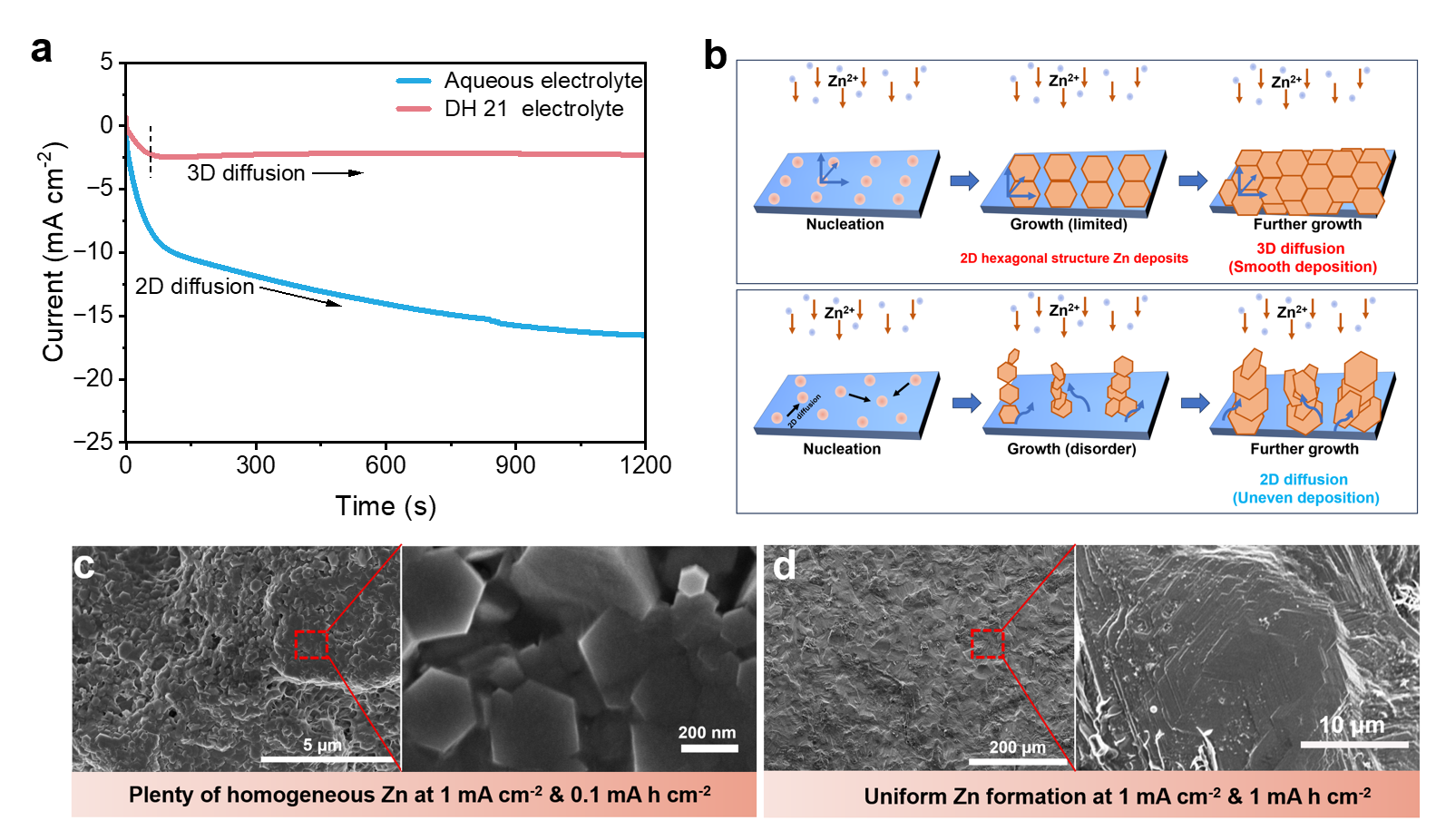


**Fig. S10** (**a**) The CA curves of Zn plate tested in both electrolytes at a fixed voltage of -150 mV. (**b**) 3D diffusion and chaotic 2D diffusion in DH21 electrolyte (upper) and aqueous electrolyte (underpart)

CA characterization was conducted on Zn//Zn symmetric cells to verify the mechanism of Zn deposition behavior. The variation in current density with time at a constant potential in CA results can sensitively reflect the nucleation process and surface change. During the rampant 2D diffusion process, the absorbed ions laterally diffuse along the surface to find the most energetically favorable sites for charge transfer. 3D diffusion implies that Zn^2+^ absorbed on the surface appear to be locally reduced to Zn^0^ with constrained 2D surface diffusion. As shown in Fig. S9, under a constant voltage of −150 mV, the current curve of Zn in DH21 electrolytes is almost unchanged and keep stable after short 2D diffusion, reflecting a constant 3D diffusion process after nucleation. A linear increase curve of Zn in aqueous electrolyte demonstrates rampant 2D diffusion process and uneven dendrite growth because of the “tip effect”and side reaction that preferentially accumulate into the higher active sites and grow vertically.


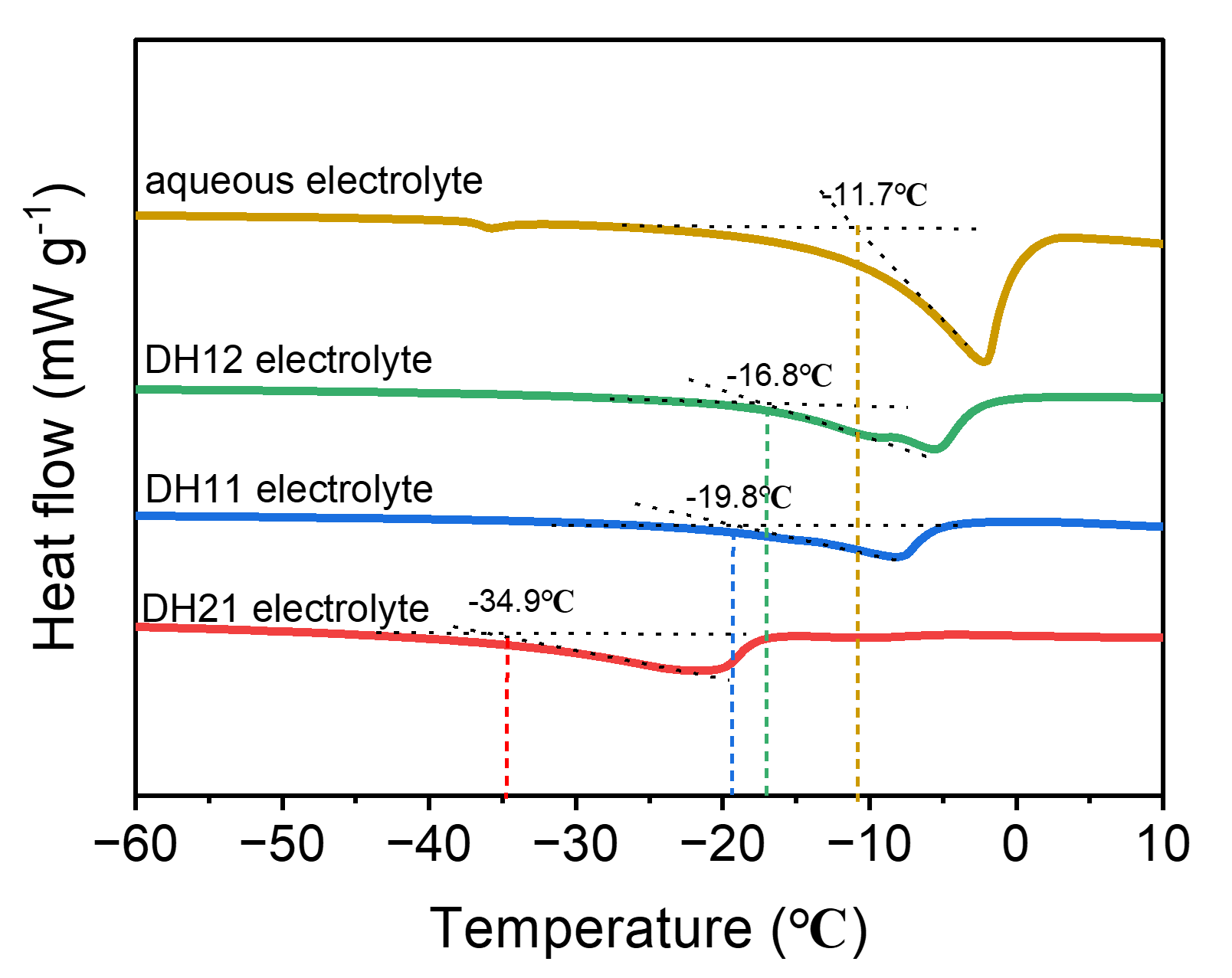


**Fig. S11** Differential scanning calorimetry (DSC) curves of different electrolytes


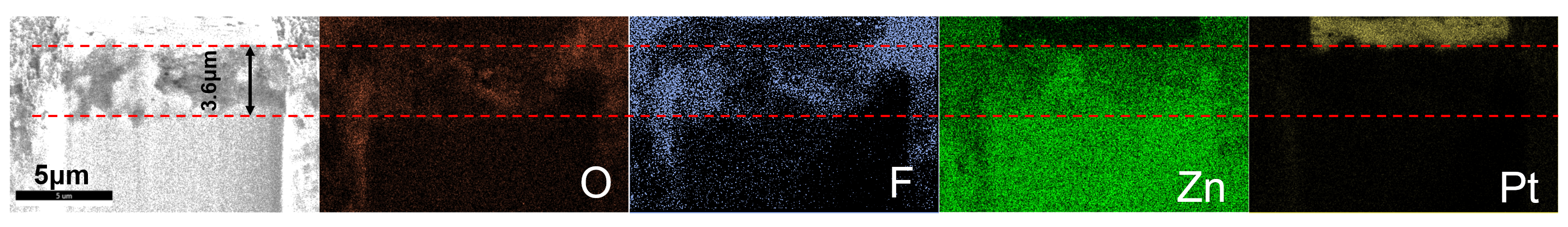


**Fig. S12** FIB-SEM images and corresponding EDX mapping of the Zn anode (the Zn electrode after 20 cycles at 0.2 mA cm^-2^, 0.2 mA h cm^-2^ in DH21 electrolyte. To protect the SEI layer from incurring electron/ion beam induced damage, the Zn electrode was coated with a Pt/C protective layer)


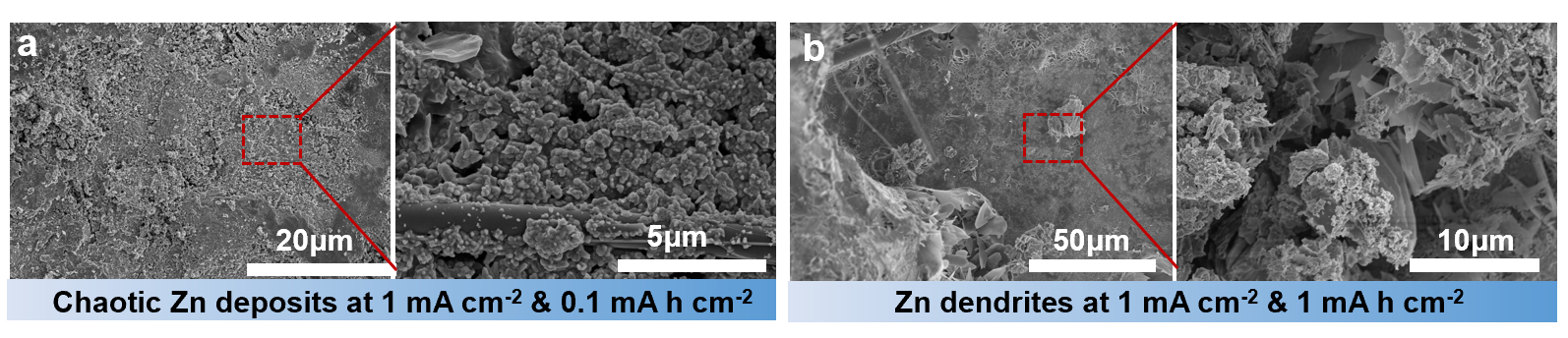
 **Fig. S13** SEM images of different Zn deposition capacities in aqueous electrolyte


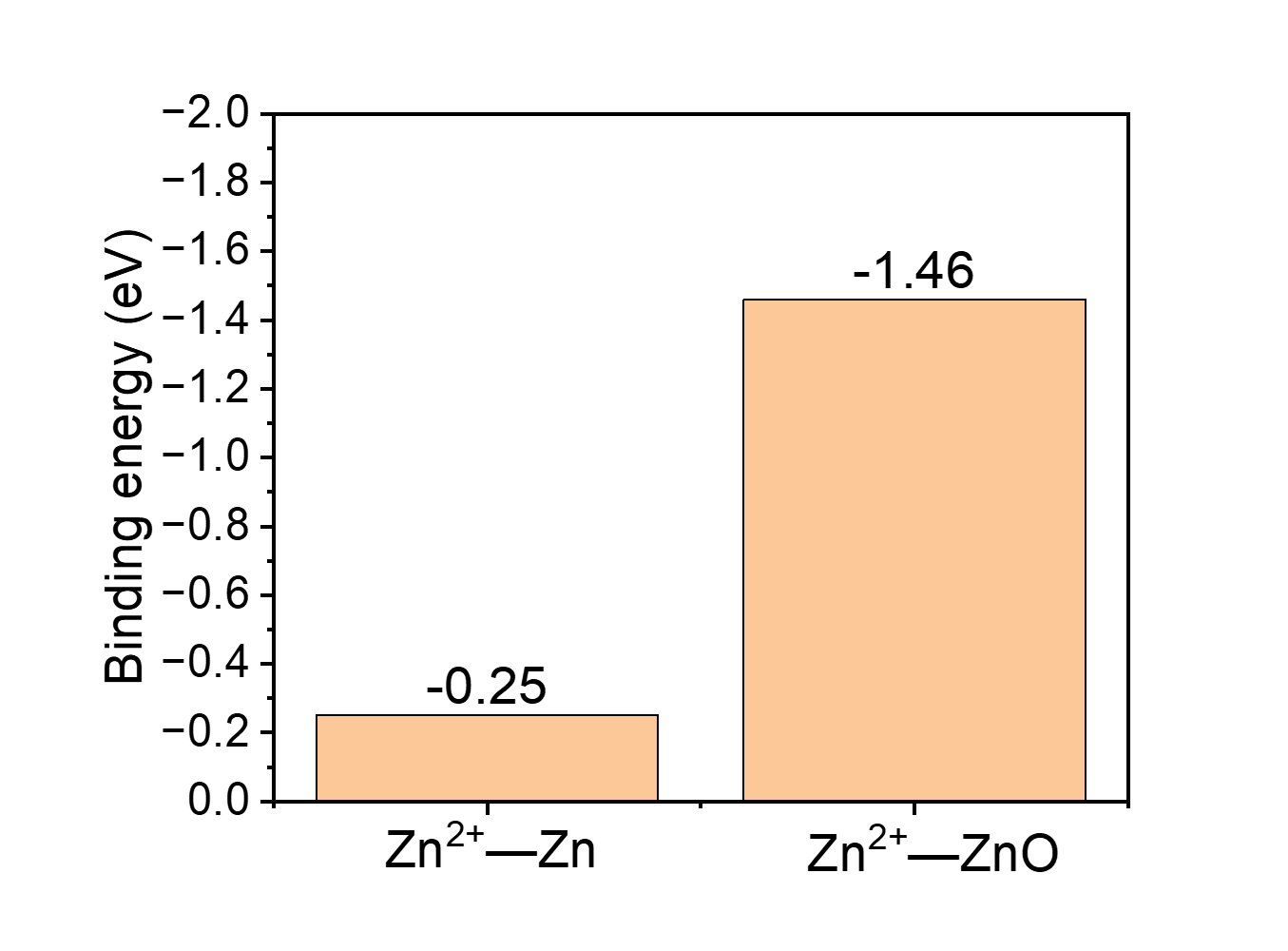


**Fig. S14** The corresponding binding energy of Zn^2+^ on bare Zn and ZnO [S4]


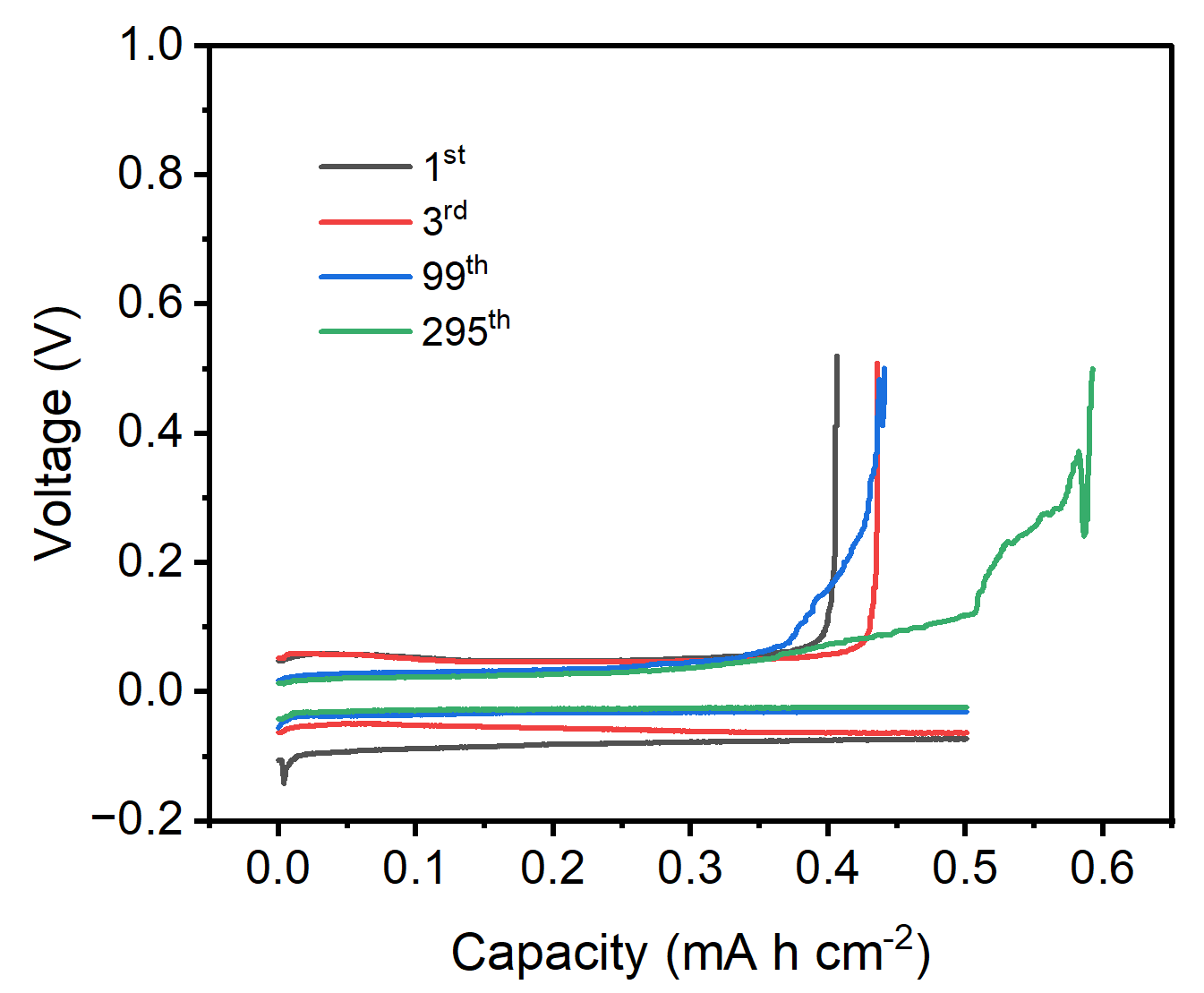


**Fig. S15** The corresponding voltage profiles of Zn plating/stripping on Cu foil at 1.0 mA cm^−2^ with 0.5 mA h cm^−2^ in aqueous electrolyte


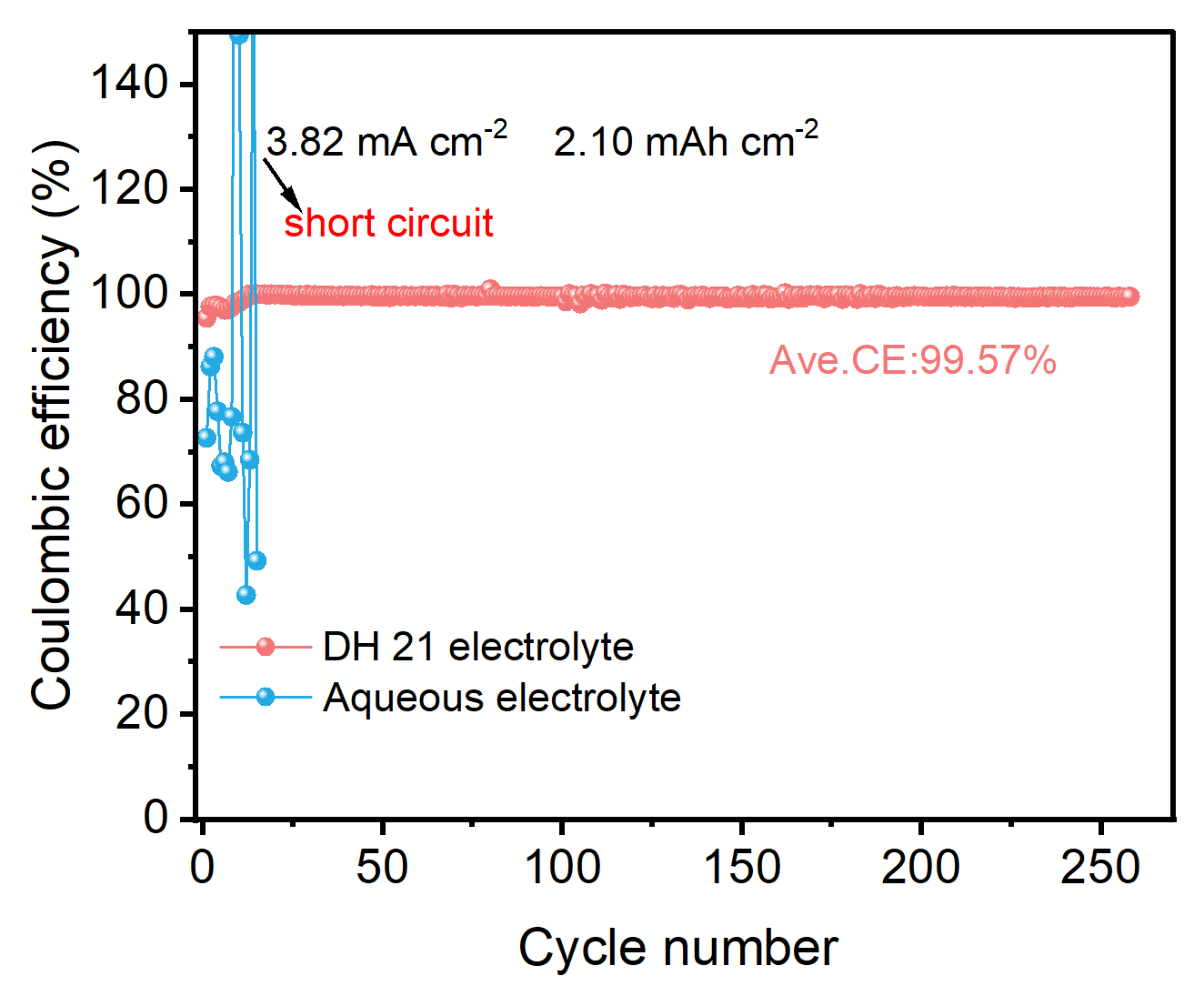


**Fig. S16** Average Coulombic efficiencies of Zn//Cu asymmetric cells with both electrolytes at 3.82 mA cm^-2^ with 2.10 mAh cm^-2^


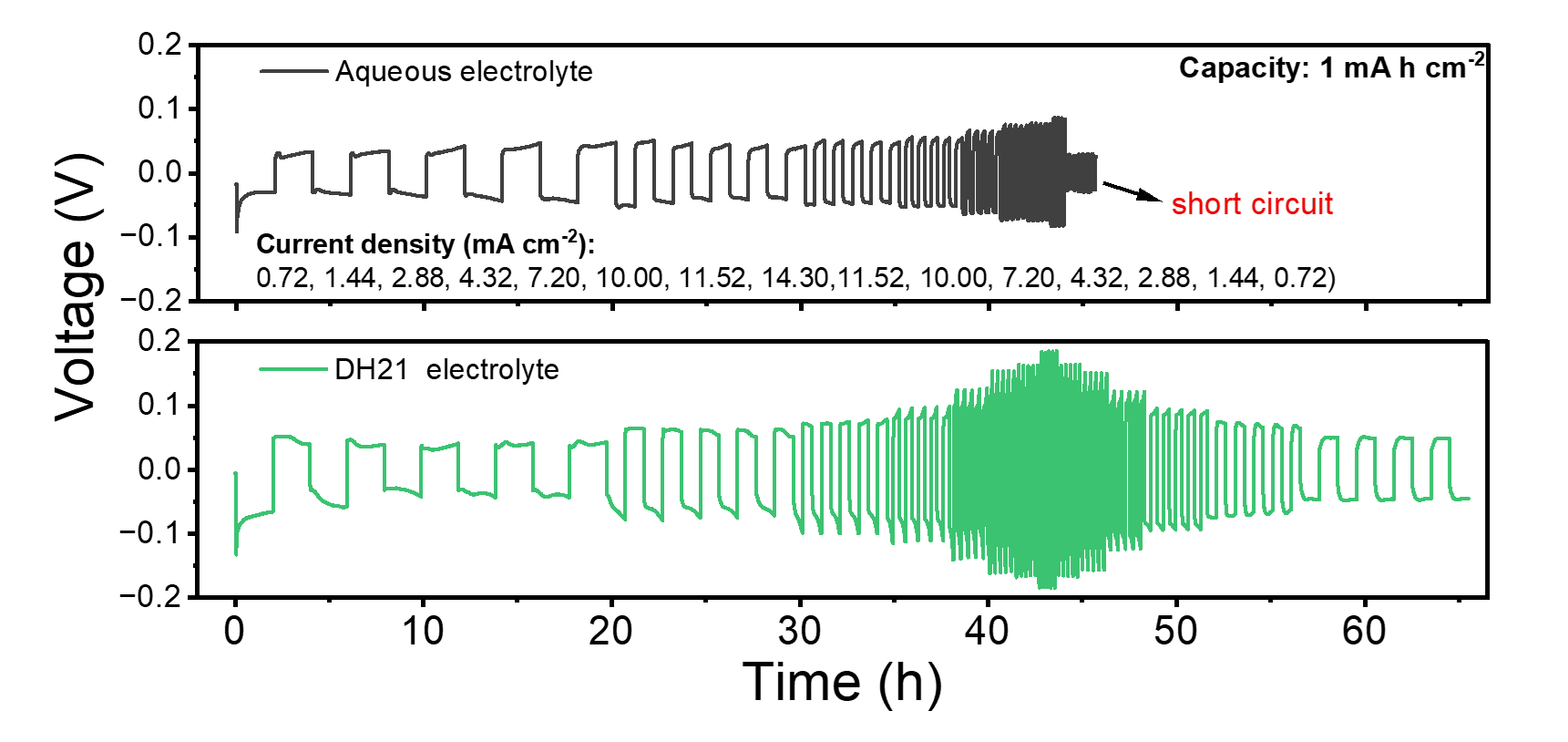


**Fig. S17** Rate performance at a fixed capacity of 1 mAh cm^−2^ of Zn//Zn symmetric cells in both electrolytes (5 cycles for each current density)


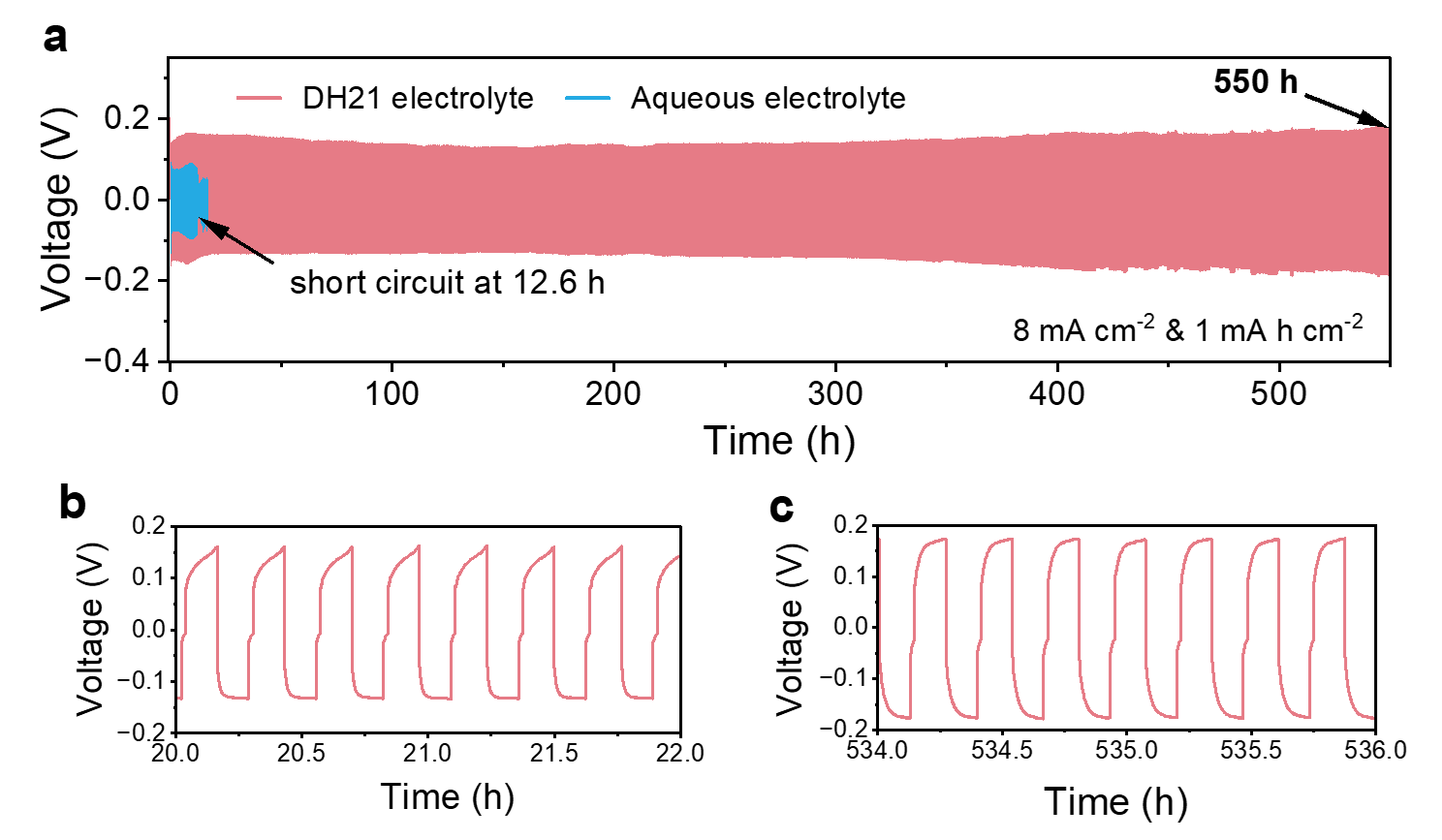


**Fig. S18** (**a**) Long-term galvanostatic Zn plating/stripping in Zn//Zn symmetric cells in both electrolytes at 8 mA cm^–2^ with a specific capacity of 1 mAh cm^–2^. (**b-c**) Locally enlarged image of Zn//Zn symmetric cells in DH21 electrolyte


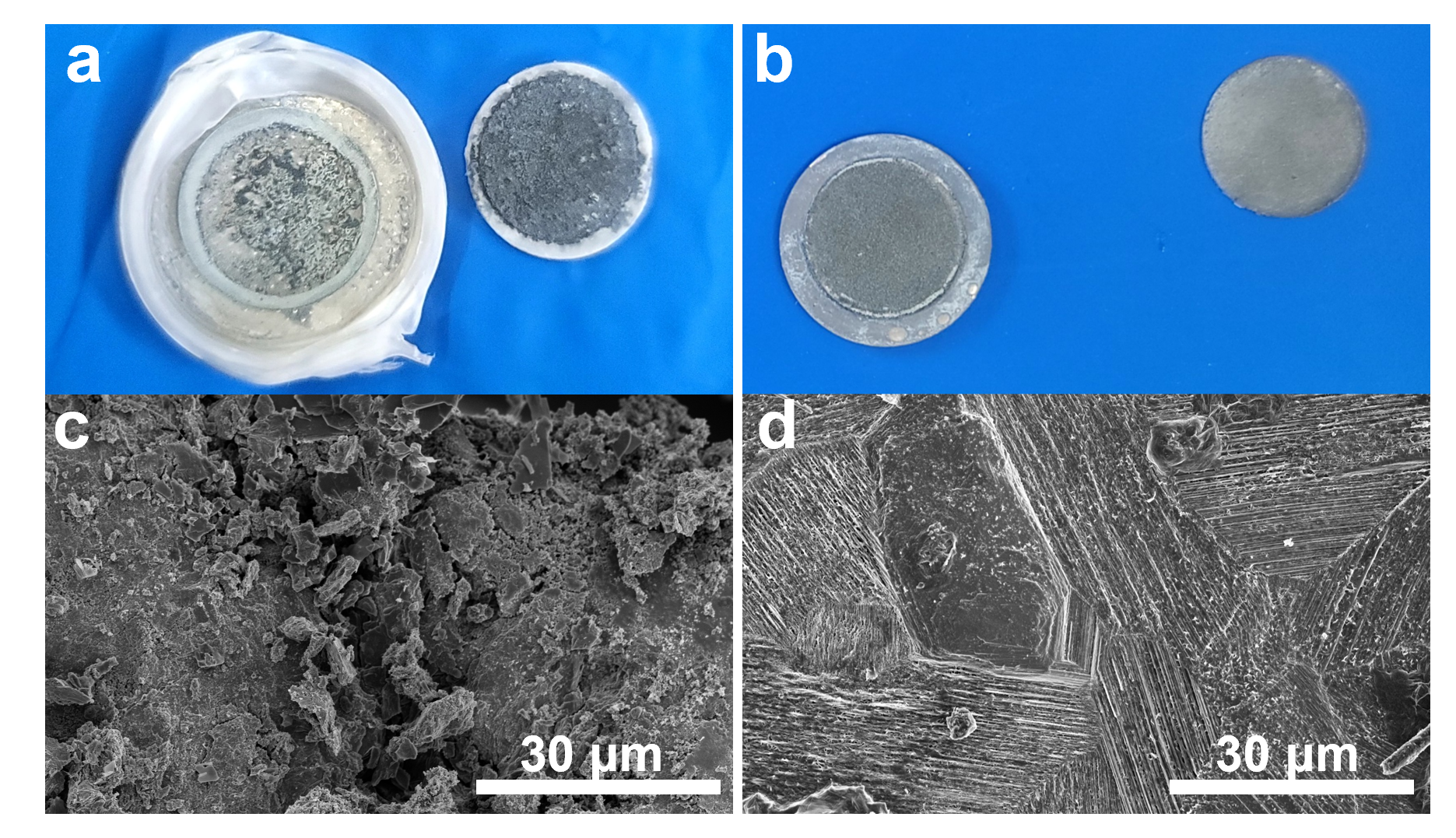


**Fig. S19** Optical photographs and SEM images of Zn after cycle at 1 mA cm^-2^ with 1 mA h cm^-2^ for 100 cycles in (**a, c**) aqueous electrolyte, and (**b, d**) DH 21 electrolyte

SEM images were performed on both electrolytes (**Fig. S19**). In aqueous electrolyte, due to the side reaction and uneven Zn^2+^ flow, the deposition mode of Zn^2+^ on the electrode surface is random and loose, which makes it easy to grow dendrites to puncture the separator and cause short circuit of the cell. In DH21 electrolyte, Zn^2+^ tends to form dense and uniform Zn deposits with the help of pDOL molecules.


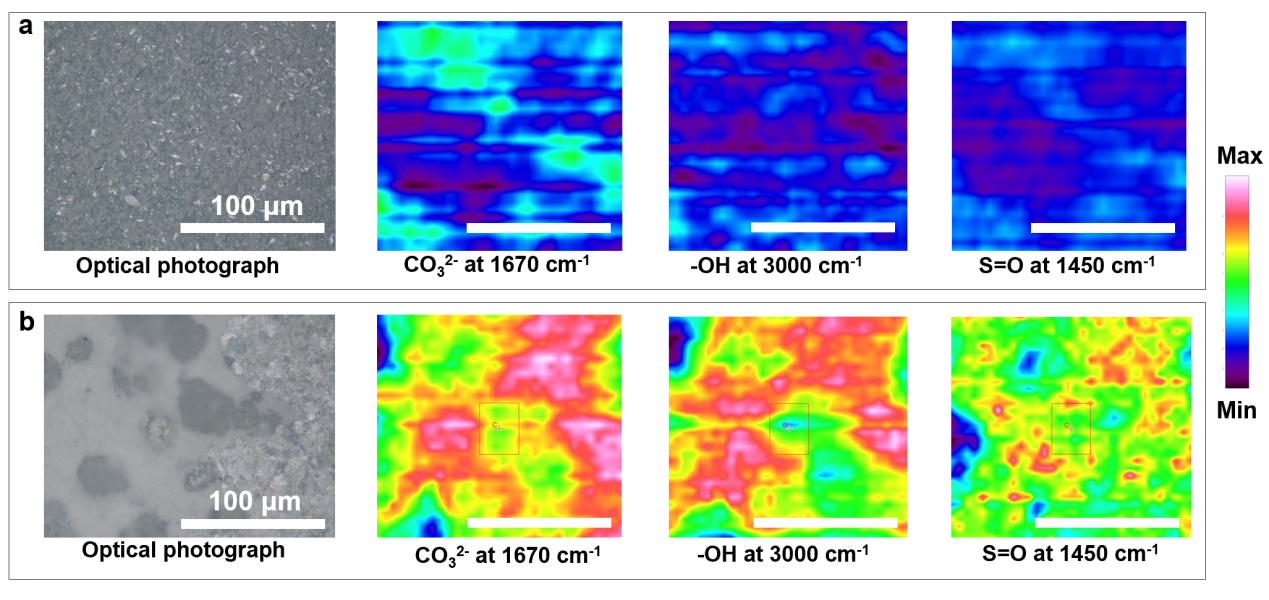


**Fig. S20** Optical photographs and Micro-FTIR mapping of Zn interface after circulation in (**a**) DH21 electrolyte, and (**b**) aqueous electrolyte


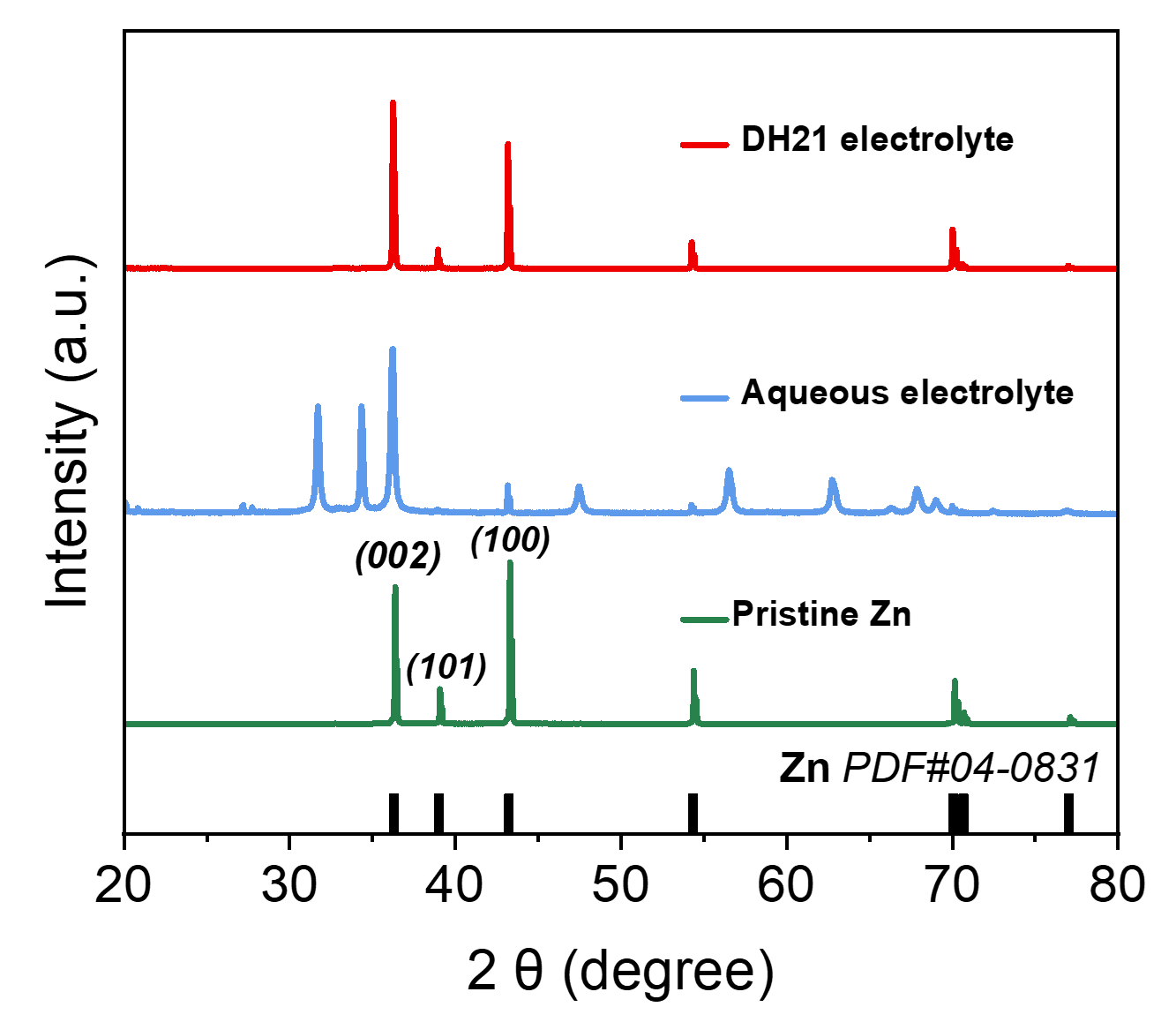


**Fig. S21** XRD patterns of pristine and cycled Zn in both electrolytes


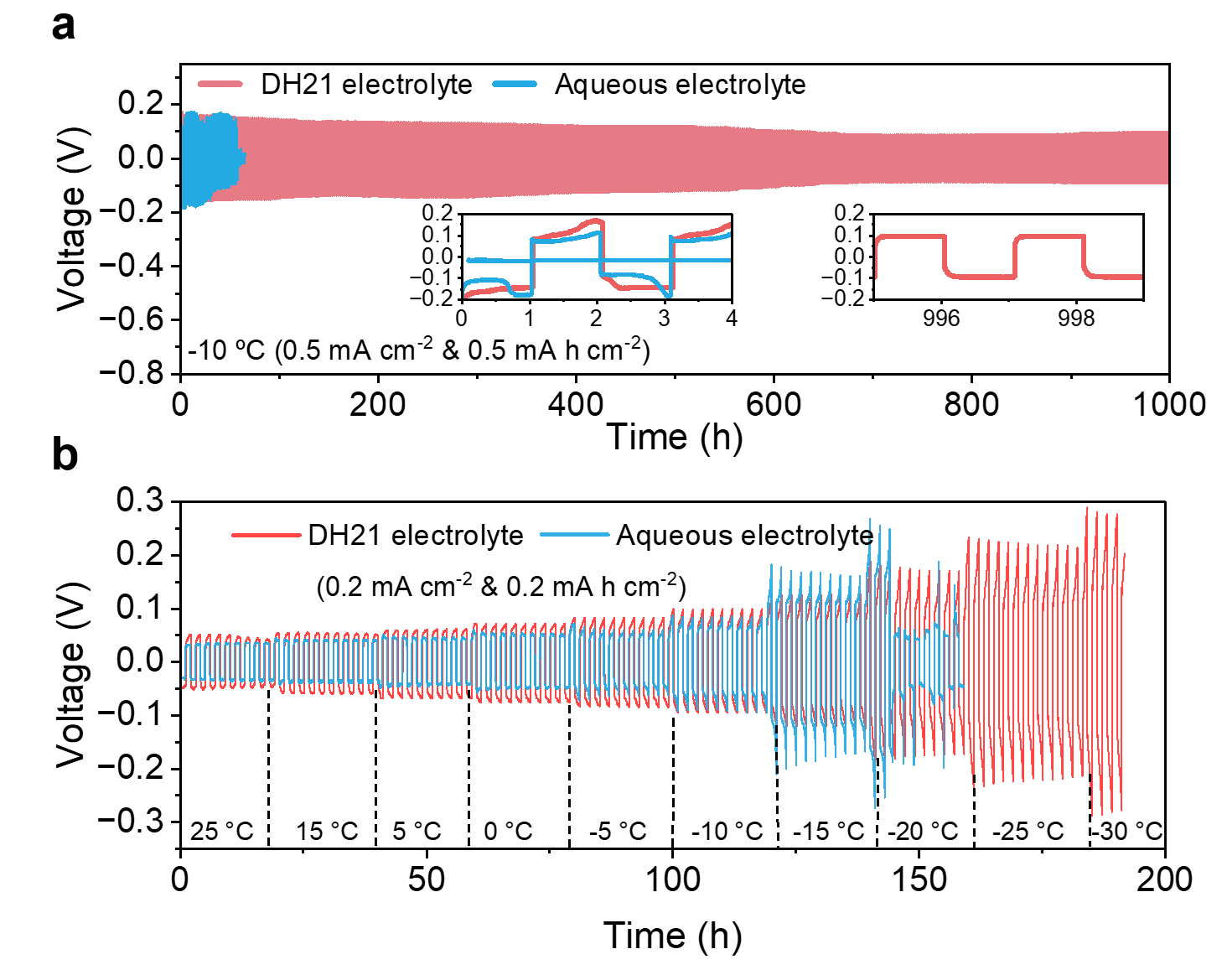


**Fig. S22** (**a**) Long-term galvanostatic Zn plating/stripping in Zn//Zn symmetric cells in both electrolytes at 0.5 mA cm^–2^ with a specific capacity of 0.5 mAh cm^–2^ at -10 ºC. (**b**) Zn//Zn symmetrical cells were cycled at different temperatures with both electrolytes


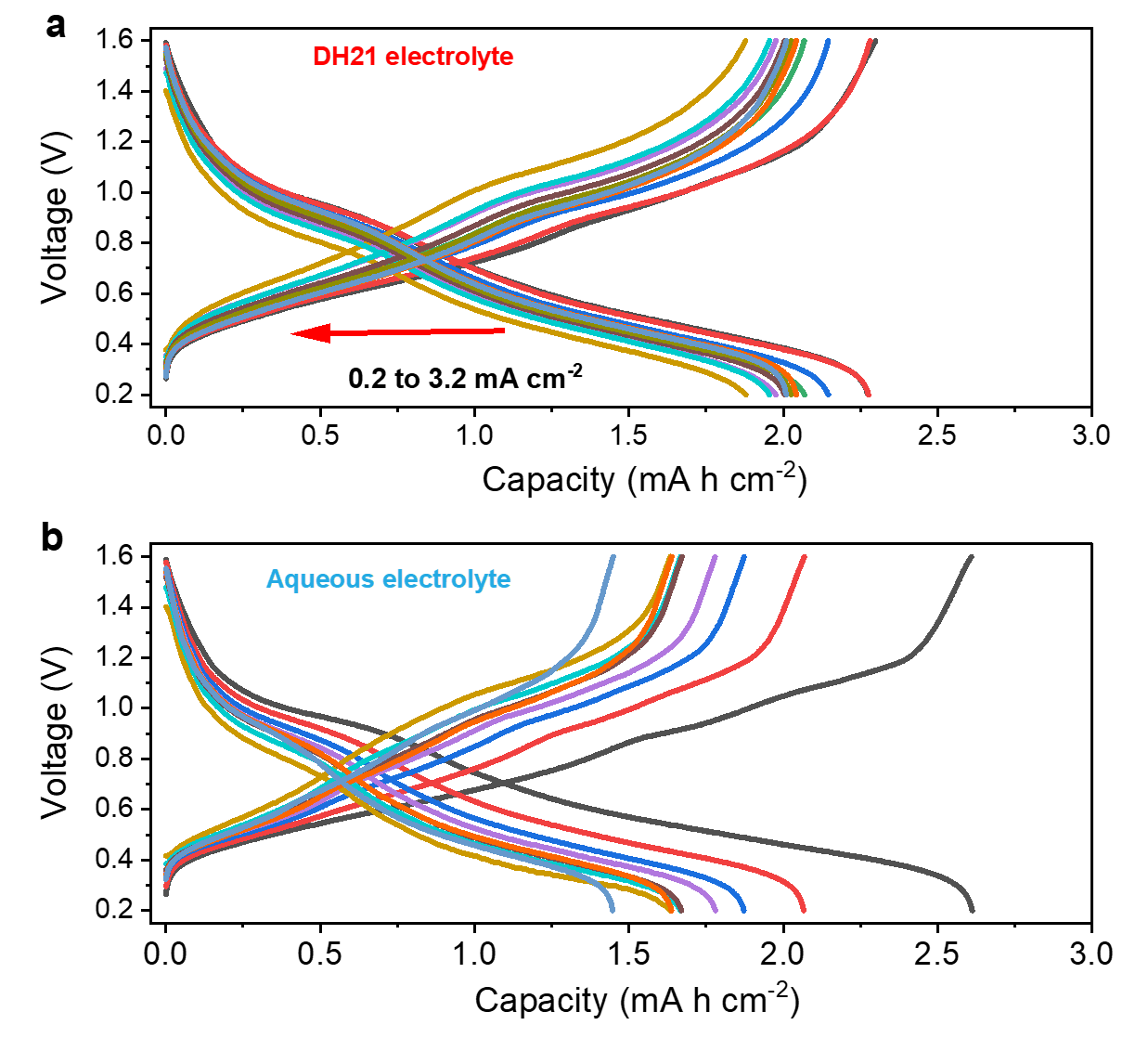


**Fig. S23** Voltage-capacity curves of Zn//VO_2_ full cells in (**a**) DH21 electrolyte, and (**b**) aqueous electrolyte


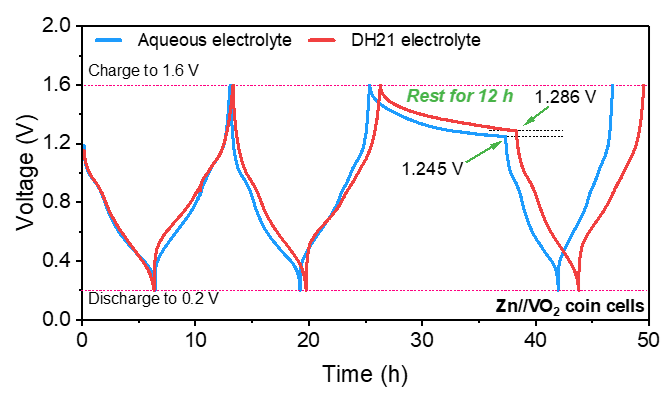


**Fig. S24** Voltage changes of Zn//VO_2_ full cells with both electrolytes at current density of 0.5 mA cm^-2^


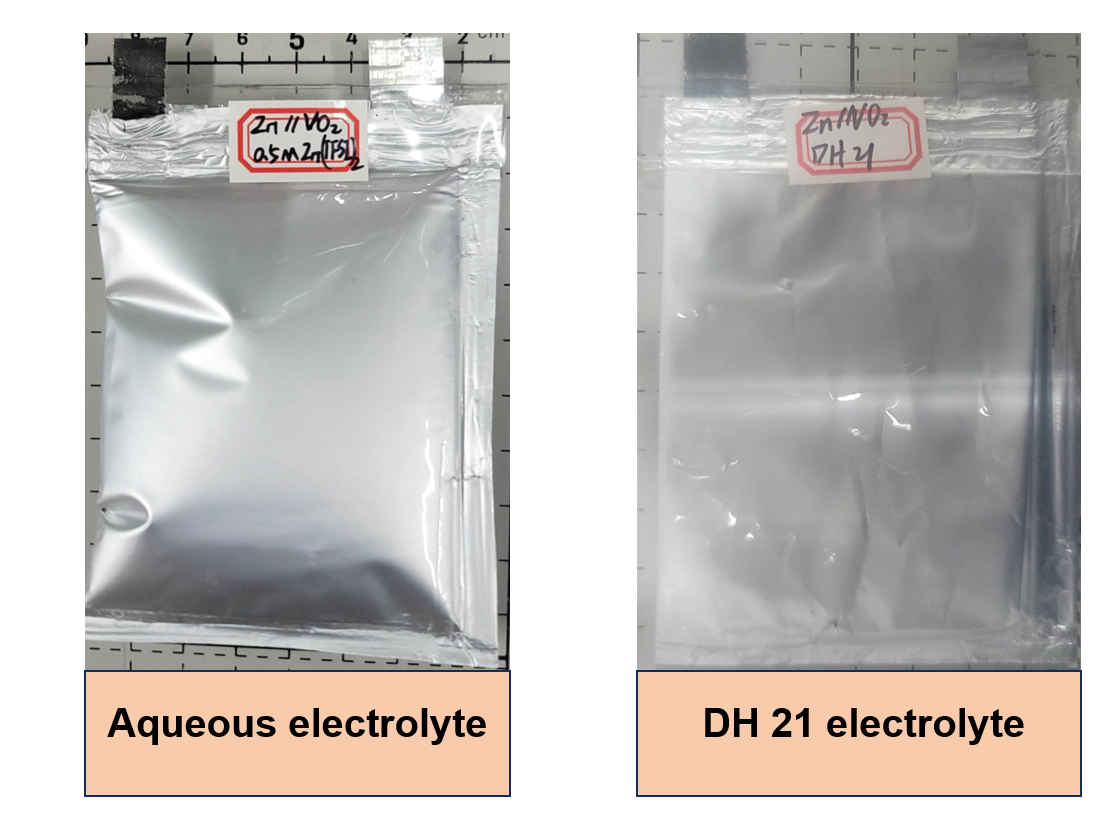


**Fig. S25** Bulges of pouch batteries after circulating in both electrolytes

In aqueous electrolyte, the pouch cell just works for 59 cycles, and then the cell fails and severe bulges occur. But for DH21 electrolyte, the Zn//VO_2_ pouch cell assembled in the lean liquid state (E/C=41 mL (A h)^-1^) has a capacity retention ratio of 92% after 600 cycles, and no significant bulges are observed.

**Table S1** Fit results of EIS with different temperatures in different electrolytes

| **Temperature**  **(℃)** | **R (Aqueous electrolyte) (Ω)** | **R (DH12 electrolyte) (Ω)** | **R (DH11 electrolyte) (Ω)** | **R (DH21 electrolyte) (Ω)** |
| --- | --- | --- | --- | --- |
| 20 | 1,432.8 | 423.5 | 346.8 | 207.3 |
| 25 | 946.1 | 252.7 | 192.4 | 123.4 |
| 30 | 608.1 | 209.5 | 138.2 | 113.6 |
| 35 | 412.6 | 110.7 | 102.9 | 94.9 |
| 40 | 277.0 | 85.8 | 73.2 | 89.2 |
| 45 | 259.6 | 34.3 | 47.2 | 70.4 |

**Table S2** Performance of lifetime and CPC of Zn//Zn symmetric cells compared with recent reports co-solvent electrolytes

| **Number** | **electrolyte** | **Current (mA cm^-2^)** | **Capacity (mA h cm^-2^)** | **Life time (h)** | **CPC (mAh cm^-2^)** | **Refs.** |
| --- | --- | --- | --- | --- | --- | --- |
| 1 | 50% PC-sat | 1 | 0.5 | 1600 | 1600 | J. Am. Chem. Soc. 2022, 144, 16, 7160–7170 |
| 2 | M56 | 5 | 5 | 1000 | 5000 | J. Am. Chem. Soc. 2023, 145, 41, 22456–22465 |
| 3 | NMP-H_2_O | 1 | 1 | 550 | 550 | Adv. Energy Mater. 2022, 12, 2103231. |
| 4 | Zn(OTf)_2_-ace | 1 | 1 | 900 | 900 | Angew. Chem. Int. Ed. 2024, 63, e202317302. |
| 5 | TEP/H_2_O BPE | 0.2 | 0.2 | 6000 | 1200 | Adv. Mater. 2024, 36, 2304426. |
| 6 | DES50 | 1 | 1 | 2000 | 2000 | Adv. Mater. 2024, 36, 2306546. |
| 7 | FEC+H_2_O | 4 | 1 | 1000 | 4000 | Angew. Chem. 2023, 135, e202216934 |
| 8 | DES-4(EG) | 0.5 | 0.5 | 2000 | 1000 | Angew. Chem. 2022, 134, e202206717 |
| 9 | 2-propanol/water | 1 | 1 | 3000 | 3000 | Adv. Mater. 2022, 34, 2207344. |
| 10 | HDES | 0.5 | 0.5 | 4000 | 2000 | Energy Environ. Sci., 2023,16, 2540-2549 |
| **11** | **DH21** | **1** | **1** | **8200** | **8200** | **This work** |
| **12** | **DH21** | **8** | **1** | **600** | **4800** | **This work** |

**Table S3** Performance of life time and ACE of Zn//Cu or Zn//Ti asymmetric cells compared with recent reports co-solvent electrolytes

| **Number** | **electrolyte** | **Current (mA cm^-2^)** | **Capacity (mA h cm^-2^)** | **Life time (h)** | **ACE (100%)** | **Refs.** |
| --- | --- | --- | --- | --- | --- | --- |
| 1 | 50% PC-sat | 1 | 0.5 | 500 | 99 | J. Am. Chem. Soc. 2022, 144, 16, 7160–7170 |
| 2 | M56 | 1 | 1 | 300 | 99 | J. Am. Chem. Soc. 2023, 145, 41, 22456–22465 |
| 3 | NMP-H_2_O | 1 | 0.5 | 1000 | 99 | Adv. Energy Mater. 2022, 12, 2103231. |
| 4 | Zn(OTf)_2_-ace | 1 | 1 | 400 | 98 | Angew. Chem. Int. Ed. 2024, 63, e202317302. |
| 5 | TEP/H_2_O BPE | 1 | 1 | 200 | 99 | Adv. Mater. 2024, 36, 2304426. |
| 6 | DES50 | 1 | 1 | 25 | 98 | Adv. Mater. 2024, 36, 2306546. |
| 7 | FEC+H_2_O | 4 | 1 | 500 | 99 | Angew. Chem. 2023, 135, e202216934 |
| 8 | DES-4(EG) | 1 | 1 | 120 | 99 | Angew. Chem. 2022, 134, e202206717 |
| 9 | 2-propanol/water | 1 | 1 | 200 | 99 | Adv. Mater. 2022, 34, 2207344. |
| 10 | HDES | 1 | 0.5 | 1000 | 99 | Energy Environ. Sci., 2023,16, 2540-2549 |
| **11** | **DH21** | **1** | **0.5** | **4200** | **99** | **This work** |

**Supplementary References**

1. A.D. Becke, Density‐functional thermochemistry. III. The role of exact exchange. J. Chem. Phys. **98**, 5648–5652 (1993). <https://doi.org/10.1063/1.464913>
2. M. Uwaha, Introduction to the BCF theory. Prog. Cryst. Growth Charact. Mater. **62**, 58 (**2016)**. https://doi.org/10.1016/j.pcrysgrow.2016.04.002
3. a) Z. Hao, Y. Zhang, Z. Hao, G. Li, Y. Lu et al., Metal Anodes with Ultrahigh Reversibility Enabled by the Closest Packing Crystallography for Sustainable Batteries. Adv. Mater. **35**, 2209985 (2023). <https://doi.org/10.1002/adma.202209985>; b) X. Zhang, J. Li, Y. Liu, B. Lu, S. Liang et al., Single [0001]-oriented zinc metal anode enables sustainable zinc batteries. Nat. Commun. **15**, 2735 (2024). <https://doi.org/10.1038/s41467-024-47101-1>
4. C. Ma, K. Yang, S. Zhao, Y. Xie, C. Liu et al., Recyclable and Ultrafast Fabrication of Zinc Oxide Interface Layer Enabling Highly Reversible Dendrite-Free Zn Anode. ACS Energy Lett. **8**, 1201 (2023). <https://doi.org/10.1021/acsenergylett.2c02735>
